# Design and synthesis of new *N-*thioacylated ciprofloxacin derivatives as urease inhibitors with potential antibacterial activity

**DOI:** 10.1038/s41598-022-17993-4

**Published:** 2022-08-15

**Authors:** Keyvan Pedrood, Homa Azizian, Mohammad Nazari Montazer, Ali Moazzam, Mehdi Asadi, Hamed Montazeri, Mahmood Biglar, Mozhdeh Zamani, Bagher Larijani, Kamiar Zomorodian, Maryam Mohammadi-Khanaposhtani, Cambyz Irajie, Massoud Amanlou, Aida Iraji, Mohammad Mahdavi

**Affiliations:** 1 Endocrinology and Metabolism Research Center, Endocrinology and Metabolism Clinical Sciences Institute, Tehran University of Medical Sciences, Tehran, Iran; 2 Department of Medicinal Chemistry, School of Pharmacy, Iran University of Medical Sciences, Tehran, Iran; 3 Department of Medicinal Chemistry, Faculty of Pharmacy, Tehran University of Medical Sciences, Tehran, Iran; 4 Department of Pharmacognosy and Pharmaceutical Biotechnology, School of Pharmacy, Iran University of Medical Sciences, Tehran, Iran; 5 Department of Medical Mycology and Parasitology, School of Medicine, Shiraz University of Medical Sciences, Shiraz, Iran; 6 Cellular and Molecular Biology Research Center, Health Research Institute, Babol University of Medical Sciences, Babol, Iran; 7 Department of Medical Biotechnology, School of Advanced Medical Sciences and Technologies, Shiraz University of Medical Sciences, Shiraz, Iran; 8 Stem Cells Technology Research Center, Shiraz University of Medical Sciences, Shiraz, Iran; 9 Central Research Laboratory, Shiraz University of Medical Sciences, Shiraz, Iran

**Keywords:** Biological techniques, Computational biology and bioinformatics, Drug discovery

## Abstract

A new series of *N*-thioacylated ciprofloxacin **3a–n** were designed and synthesized based on Willgerodt–Kindler reaction. The results of in vitro urease inhibitory assay indicated that almost all the synthesized compounds **3a–n** (IC_50_ = 2.05 ± 0.03–32.49 ± 0.32 μM) were more potent than standard inhibitors, hydroxyurea (IC_50_ = 100 ± 2.5 μM) and thiourea (IC_50_ = 23 ± 0.84 μM). The study of antibacterial activity against Gram-positive species (*S. aureus* and *S. epidermidis*) revealed that the majority of compounds were more active than ciprofloxacin as the standard drug, and **3h** derivative bearing 3-fluoro group had the same effect as ciprofloxacin against Gram-negative bacteria (*P. aeruginosa* and *E. coli*). Based on molecular dynamic simulations, compound **3n** exhibited pronounced interactions with the critical residues of the urease active site and mobile flap pocket so that the quinolone ring coordinated toward the metal bi-nickel center and the essential residues at the flap site like His593, His594, and Arg609. These interactions caused blocking the active site and stabilized the movement of the mobile flap at the entrance of the active site channel, which significantly reduced the catalytic activity of urease. Noteworthy, **3n** also exhibited IC_50_ values of 5.59 ± 2.38 and 5.72 ± 1.312 µg/ml to inhibit urease enzyme against *C. neoformans* and *P. vulgaris* in the ureolytic assay.

## Introduction

Bacterial infections, predominantly caused by Gram-positive and Gram-negative organisms, are among the world’s principal causes of morbidity and even mortality both in the community and hospital^1^. Albeit there are a considerable number of antibacterial agents to eliminate or suppress the growth of these pathogens^2–4^, there is still great failure to control the bacterial infectious owing to multi-drug resistant (MDR) pathogens that are extraordinarily resistant to the routine antimicrobial agents^5^. Methicillin-resistant *S. epidermidis* (MRSE), methicillin-resistant *S. aureus* (MRSA), vancomycin-resistant *S. aureus* (VRSA), producing *E.* *coli*, extended-spectrum β-lactamase (ESBL) drug-resistant TB (DR-TB) are among the examples of the evolved drug-resistant pathogens^6–8^. The statistics reveal about 700,000 deaths are related to bacterial infection annually, and this number will increase dramatically to 10 million in 2050 if no practical solution is discovered^9,10^. As a result, the need for the synthesis of new drug-like small molecules is critical^11^. Ciprofloxacin, as the second generation of fluoroquinolones, has demonstrated to have notable activity in this regard. Furthermore, the suitable pharmacokinetic profile of fluoroquinolones and their relative clinical safety have made them a valuable therapeutic choice for various respiratory, soft tissue, and bone infections^11^.

Urease enzyme, the first enzyme ever prepared and isolated in crystalline form in 1926^12^, is a cell-surface nickel-containing enzyme available in a myriad of living things. It is also the most potent enzyme that enhances the reaction rate approximately 10^15^ times faster compared to uncatalyzed reactions^13^. The vital function of urease is the hydrolysis of urea to convert it into ammonia and carbon dioxide^14,15^. This process results in the formation of a considerable amount of ammonia through the urea hydrolysis when *Helicobacter pylori* (*H. pylori*) utilizes this enzyme to survive the harsh acidic conditions of the stomach^16^. Under these circumstances, this human pathogenic bacterium colonizes the stomach and leads to many severe gastrointestinal (GI) diseases, including gastric ulcers, gastritis, and stomach cancer^17,18^. The *H. pylori* infection is a major cause of GI problems and it is highly prevalent worldwide^19,20^. As a result, inhibition of urease activity can be construed as a favorable action to mitigate the negative effect of ureolytic bacterial infections in humans^21^. Along the same line, designing new urease inhibitors is of critical importance for approximately 50% of the world’s human population infected by such a human bacterium. Urea and thiourea derivatives^15,22,23^, compounds containing phosphate^24^, five and six-membered heterocycles, natural products, and metal complexes^25^ are the main sub-categories into which the inhibitors are divided.

Here, we report the design, synthesis, and in vitro urease inhibitory activity as well as antibacterial potential of *N-*thioacylated ciprofloxacin derivatives. In addition, in silico molecular docking and molecular dynamic simulations were performed.

## Result and discussion

### Designing

**Figure 1:**
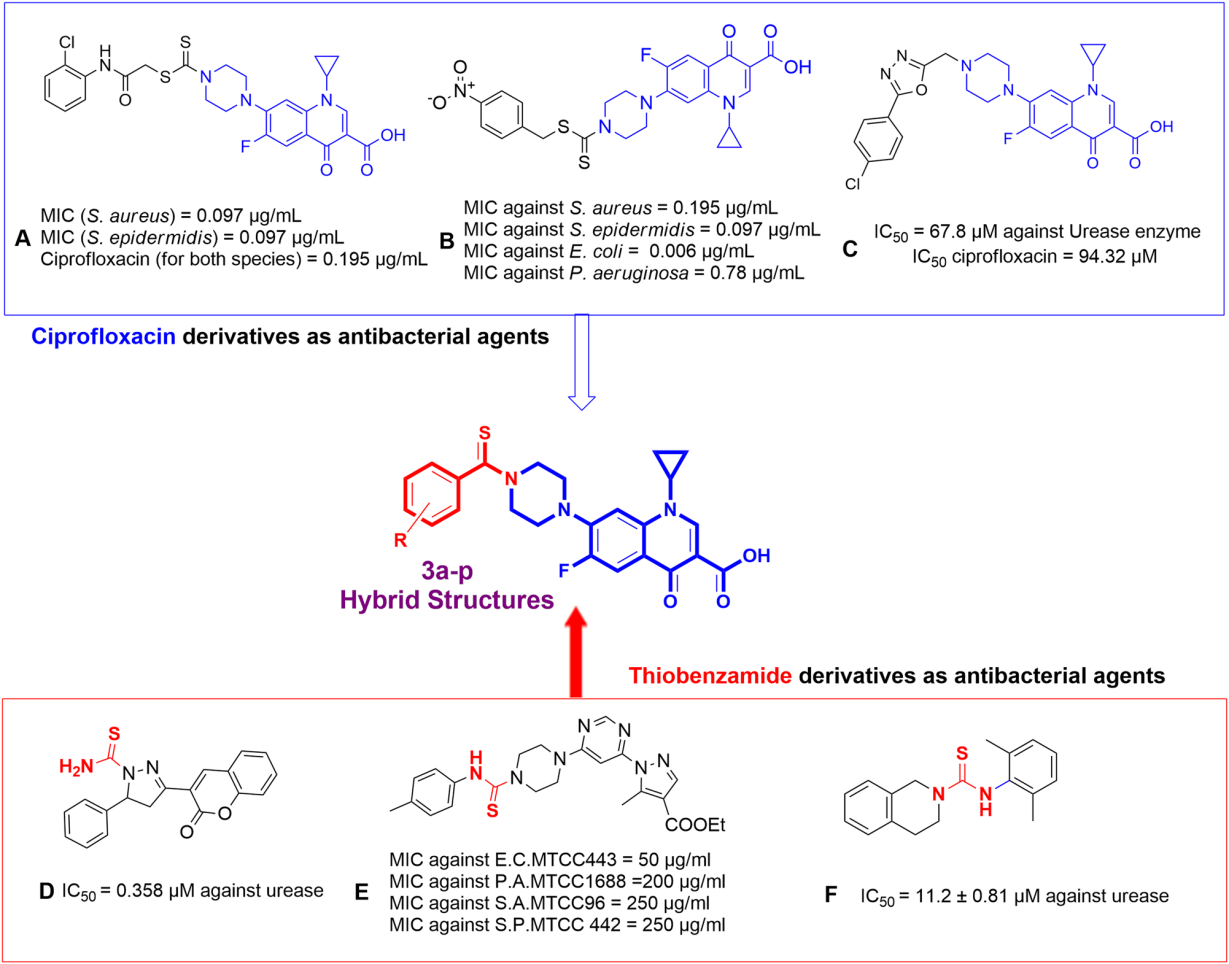
Identified representative lead candidates.

Ciprofloxacin derivatives were excessively reported as highly potent antibacterial agents^26,27^. Ciprofloxacin-dithiocarbamate hybrid bearing *ortho*-chlorine group exhibited promising effects on the standard Gram-positive bacteria (Fig. 1, compound **A**) while changing the chlorine position from *ortho* to *meta* increased the activity against Gram-negative bacteria^28^. In silico assessment also demonstrated the important role of the sulfur atom through forming hydrogen bonds with the residues of *S. aureus* DNA gyrase^28^. Compound **B** is another example of a potent antimicrobial agent with ciprofloxacin moiety. SARs reveals that a nitro substituent at the 4-position of the benzyl ring improved the antimicrobial activities compared to the rest of the synthesized derivatives. A molecular docking study of compound **B** against *E. coli* showed that the sulfur atom of the dithiocarbamate participated in two interactions with residues Asn46 and Val120, and the ciprofloxacin group also interacted with residues Arg76, Ile78, Pro79, Ile90, and Arg136 in the active site of DNA gyrase^29^. Along the same line, the urease inhibitory and antibacterial activities of the ciprofloxacin-piperazinyl derivatives were proved (Fig. 1, compound **C**)^30^. These results demonstrated the critical role of ciprofloxacin as a critical building block for the design of anti-urease agents.

Thioamides, whether cyclic analogs or open-chain structures are among the fascinating organic compounds because of their adequate stability and low toxicity^31^. Thioamide moiety showed beneficial activity against various types of bacterial^32^, and fungal infections^33^. Dixon et al. showed for the first time that acetamide is a substrate for urease with a *K*_*m*_ value of 750 (at pH 7) which was around 260-fold better than that of urea. These findings showed that such moiety properly fitted in the binding site of the urease enzyme^34^. In another study, the urease inhibitory effect of coumarinyl-pyrazolinyl-thioamide derivatives against *jack bean* urease was evaluated. The most potent derivative (Compound **D,** Fig. 1) showed significant inhibitory activities (IC_50_ = 0.000358 ± 0.000017 µM) compared to thiourea as positive control (IC_50_ = 4.720 ± 0.174 µM). A molecular docking study of compound **D** in the binding pocket of urease showed that the thioamide group forms hydrogen bonds with Ala440 and Asp494^35^. In another study, *N*-thioamide analogs of pyrazolylpyrimidine were also shown to have antibiotic activity against bacterial species (Fig. 1, compound **E**). In silico study exhibited that this moiety as hydrogen sulfide donor participated in several interactions with the binding pocket of *E. coli*, *S. aureu*s Hydrolase, and *P. falciparum* dihydrofolate reductase enzymes. Recently, N-Aryl-3,4-dihydroisoquinoline carbothioamide analogs were tested against urease and recorded IC_50_ values in the range of 11.2 to 50.6 µM compared to the standard thiourea (IC_50_ = 21.7 ± 0.34 μM). Molecular docking studies of the most potent compound (compound **F**) presented strong interactions between sulfur and two Ni co-factors. Also, the nitrogen of the thioamide linker participated in H-bound interaction with the residue of the binding site^36^. Mentioned point inspired the use of aryl-thioamide moieties as an important functional group in the design of the new urease inhibitors.

Inspired by all these findings, a new series of *N-*thioacylated ciprofloxacin **3a–n** were designed as antimicrobial and anti-ureolytic agents. It was proposed that ciprofloxacin as an elegant skeleton provide the antimicrobial activity of derivatives while thioamide moiety implemented the condition so that compounds could properly fit in the active site of urease and the improvement of anti-urolytic activities might be seen. Furthermore sulfur might afford better interactions with critical Ni (I) and Ni (II) coordinated with His519, His545, Lys490, His407, His409, Asp633, and Lys490 residues^37^. Compounds were synthesized using a facile and straightforward method and were evaluated against Gram-positive and Gram-negative bacteria. All the mentioned derivatives, besides, were evaluated for their in vitro urease inhibitory activities. Along the same line, in silico induced fit docking and molecular dynamic studies were performed to investigate the interaction, orientation, and conformation of the compounds with the best inhibitory activity over the active site of *jack bean* (*JB*) urease.

### Chemistry

**Figure 2:**
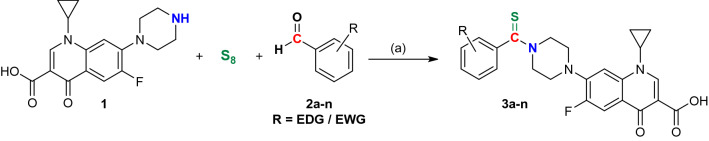
Outline for the synthesis of *N-*thioacylated ciprofloxacin derivatives **3a–n**. Reagents and conditions: (a) DMSO, 50 °C, 4–8 h.

The synthetic method employed to prepare *N-*thioacylated ciprofloxacin derivatives **3a–n** is depicted in Fig. 2. Commercial ciprofloxacin **1** was submitted to thioamidation with the equivalent amount of various aldehydes **2a–n** in the presence of sulfur (S_8_) to give the corresponding final products **3a–n**, respectively. The latter compounds were fully characterized by ^1^H NMR, ^13^C NMR, FT-IR, and elemental analysis.

**Figure 3:**
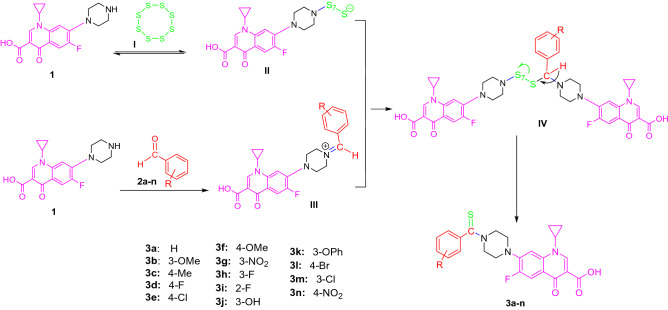
A plausible mechanism for the preparation of **3a**.

Kale et al. developed a facile Willgerodt–Kindler-type reaction for preparing thioamides under catalyst‐free conditions at room temperature or 120 °C in DMSO^38^. The plausible mechanism for this Willgerodt–Kindler reaction is proposed in Fig. 3. In this pathway, ciprofloxacin molecule **1** is featured in two roles. Initially, the elemental cyclooctasulfur (S_8_) **I** undergoes a nucleophilic attack by the secondary amine group on the ciprofloxacin **1**, which results in the cleavage of S–S bond, and reversible formation of polysulfide anion **II**. In the meantime, the different aldehyde **2a-n** react with another molecule of ciprofloxacin** 1** to form the intermediate iminium ion **III** by removal of the hydroxyl group. The reaction between the intermediates **II** and **III** subsequently leads to the formation of **IV,** which experiences oxidation to yield the desired product **3**.

### In vitro inhibitory activity of *N-*thioacylated ciprofloxacin derivatives against *JB* urease

All the *N-*thioacylated ciprofloxacin derivatives **3a–n** were screened against *JB* urease enzyme. The obtained results revealed that (Table 1) most of the compounds showed significant inhibition against urease with IC_50_ values of 2.05 to 32.49 µM compared to hydroxyurea and thiourea as reference inhibitors with an IC_50_ value of 100 and 23 µM, respectively.

**Table 1:** The urease inhibitory activity of the synthesized compounds **3a–n**.


Compound	R	IC_50_ (µM)^a^
**3a**	H	10.61 ± 0.09
**3b**	3-OMe	17.02 ± 0.16
**3c**	4-Me	20.63 ± 0.19
**3d**	4-F	2.94 ± 0.04
**3e**	4-Cl	6.55 ± 0.05
**3f**	4-OMe	21.36 ± 0.08
**3g**	3-NO_2_	5.87 ± 0.07
**3h**	3-F	4.08 ± 0.03
**3i**	2-F	14.97 ± 0.16
**3j**	3-OH	19.60 ± 0.17
**3k**	3-OPh	32.49 ± 0.32
**3l**	4-Br	8.21 ± 0.06
**3m**	3-Cl	12.24 ± 0.24
**3n**	4-NO_2_	2.05 ± 0.03
Hydroxyurea	–	100.0 ± 2.5
Thiourea	–	23 ± 0.84

^a^Values are the mean ± SEM. All experiments were performed at least three times.

In particular, the 4-nitro derivative (**3n**) was the most promising urease inhibitor of this series (IC_50_ = 2.05 µM). Further investigations illustrated that the compound **3d** (IC_50_ = 2.94 µM) containing 4-fluoro substitution, which is also an electron-withdrawing group was another potent inhibitor in this series. Other *para*-substituted derivatives such as **3e** (R = 4Cl, IC_50_ = 6.55 µM) and **3l** (R = 4Br, IC_50_ = 8.21 µM) even demonstrated better inhibitory activity compared to reference inhibitors. With these reports in hand, it was estimated that the presence of electron-withdrawing groups at the *para* position is a potential structural point for urease inhibition activity.

Afterward, the effect of the same substitutions on the *meta*-position was explored. In testing the compounds **3g** and **3h**, it was shown that 3-nitro substitution (**3g**) has less effect on empowering the urease inhibitory potential than the 3-fluoro derivative (**3h**). A plausible explanation for such a difference can be drawn from the variation of the groups’ steric hindrance, in a way that the more bulky group 3-nitro’s effect was lower compared to 3-fluoro (which is not much larger than hydrogen). The compound **3k** with the phenoxy group in *meta*-position had the lowest effect due to the substitution’s considerable steric hindrance. This compound was the only compound that showed less activity than thiourea as the standard drug.

The importance of steric hindrance and its effect on the urease inhibition potential of the compounds was also proved by comparing *ortho*-substituted one (**3i**) with others mentioned above. Obviously, *ortho*-substitutions provide more hindrance than the *meta*- and *para*-substituents. Therefore, the presence of bulky groups in this position has a higher negative impact. The hypothesis was strengthened further by the inhibitory effect reports of **3e** and **3m** with Cl at *para*, and *meta* position, respectively.

Again, to prove the positive effect of the electron-withdrawing ability of substitutions and the negative influence of steric hindrance, a comparison between compounds bearing 4-Cl and 4-Br (respectively **3e** and **3l**) showed that the *para*-chloro-substituted inhibitors had a higher potency than the *para*-bromo-substituted one. Plus, the presence of electron-donating groups, namely hydroxyl, methoxy, and methyl in either position decreased the inhibition potential compared to **3a**.

### Antimicrobial activity and structure–activity relationships (SAR) study

Antibacterial activity of the synthesized compounds **3a–n** were determined according to the agar dilution methods for Gram-positive bacteria strains (*Staphylococcus aureus* ATCC 6538 and *Staphylococcus epidermidis* ATCC 12228) and Gram-negative bacteria strains (*Escherichia coli* ATCC 8739 and *Pseudomonas aeruginosa* ATCC 9027). Table 2 shows the minimum inhibitory concentrations (MICs) values of the target compounds in comparison with ciprofloxacin as the standard drug.

**Table 2:** Structure and antibacterial activity of compounds **3a–n**.


Compound	R	MIC (µg/ml)			
		*S. aureus*	*S. epidermidis*	*P. aeruginosa*	*E. coli*
**3a**	H	0.048	0.024	6.250	0.048
**3b**	3-OMe	0.048	0.024	3.125	0.097
**3c**	4-Me	0.048	0.024	3.125	0.097
**3d**	4-F	0.097	0.048	0.781	0.097
**3e**	4-Cl	0.048	0.048	0.781	0.024
**3f**	4-OMe	0.024	0.024	3.125	0.097
**3g**	3-NO_2_	0.024	0.024	0.390	0.012
**3h**	3-F	0.048	0.097	0.048	< 0.003
**3i**	2-F	0.024	0.024	2.500	0.0195
**3j**	3-OH	0.097	0.048	0.781	0.024
**3k**	3-OPh	0.390	0.390	3.125	0.097
**3l**	4-Br	0.048	0.048	0.848	0.028
**3m**	3-Cl	0.048	0.097	6.250	0.048
**3n**	4-NO_2_	0.781	0.390	1.562	0.097
Ciprofloxacin	–	0.024	0.048	0.048	< 0.003

The screening of the antibacterial results disclosed a considerable number of compounds that displayed better activity against *S. epidermidis* in comparison with the parent drug ciprofloxacin*.* Also, some of the products showed similar activity to ciprofloxacin against *S. aureus* such as **3f.** (R = 4-OMe)*,* **3g** (R = 3-NO_2_)*,* and **3i** (R = 2-F). Noteworthy, compound **3h** (R = 3-F) showed similar activity to ciprofloxacin against *P. aeruginosa* and *E. coli* with MIC values of 0.048 and < 0.003 µg/ml, respectively. To better understand the SARs, synthetic compounds were divided into four groups based on tested strains.

Assessment on type and position of halogen-substituted groups against *S. aureus* demonstrated the following other of potency R = 2-F (**3i**) > R = 3-F (**3h**)–R = 3-Cl (**3m**)–R = 4-Cl (**3e**) > R = 4-Br (**3l**) > R = 4-F (**3d**). It seems that *ortho* fluorine substitution had the most dominant role in anti-*S. aureus* activity. On the other hand, as can be seen in **3b**, **3c**, **3j**, and **3k,** electron-donating groups had destructive effects against *S. aureus*. The exception in this trend came back to **3f** containing 4-OMe with a MIC value of 0.024 µg/ml. Investigation of the nitro as a strong electron-withdrawing substitution showed that the **3g** bearing *ortho* group had superior activity compared to the *para* counterpart (**3n**).

In the case of *S. epidermidis,* similarly, **3i** (R = 2-F) exhibited significant inhibitory activity followed by *para*-halogen substituted groups (**3d**, **3e,** and **3l** with MIC = 0.048 µg/ml) and *ortho* ones (**3m** and **3h** with MIC = 0.097 µg/ml). It can be easily understood that position of halogen had a more important role compared to the type of substitutions. In comparison with the parent drug ciprofloxacin, *S. epidermidis* had lower activity in the presence of **3b** (R = 3-methoxy), and **3c** (R = 4-methyl) as the electron-donating groups; however, these derivatives exhibited lower potency on the other tested species. In comparison with the other electron-donating derivatives, the significant advantage of compound **3f** was that it had a serious impact not only on *S. epidermidis,* but also on *S. aureus.* Similar to *S. aureus meta-*nitro moiety (**3g**) possessed better anti-*S. epidermidis* activity compared to *para-*nitro derivative (**3n**)*.*

From the screening data, it was revealed that just **3h** (3-F) demonstrated similar activity to ciprofloxacin in comparison with the rest of the derivatives against *P. aeruginosa*. It was proposed that its small size with moderate lipophilicity plus weak electron-withdrawing properties had the most critical role against *P. aeruginosa.* Other halogenated compounds did not generally show a significant potency against *P. aeruginosa* in comparison with **3h**. The other potent derivative in this group was **3g** > **3e** > **3l**.

Noteworthy these compounds showed better activity against *E. coli* with MIC values ranging from < 0.003 to 0.097 µg/ml than *P. aeruginosa* (MIC range = 0.048–6.250 µg/ml) and compound **3h** containing 3-F exhibited the best potency in this set. Although there were no statistically significant differences among the rest of the derivatives; however, in most cases halogen-substituted groups seem to slightly improve the anti-*E. coli* activity.

SARs assessment regarding all tested strains it can be seen that the substitution of the *meta*-position by nitro in the compound **3g** caused an identical effect compared to *para*-position (**3n**) on all tested bacteria*.* Compound **3f** (R = 4-OMe) bearing electron-donating group, improved the effect on all tested Gram-positive bacterias. Examining the effects of fluorine on the *para*- (**3d**), *meta*- (**3h**), and *ortho*- (**3i**) positions proved that the *ortho*-position-substituted fluorine compound had the best outcome on Gram-positive bacteria while 3-F represented ciprofloxacin-like effects on the Gram-negative bacterium *P. aeruginosa* and *E. coli.* Evaluation of Gram-positive bacteria exhibited that **3f** (R = 4-OMe), **3g** (R = 3-NO_2_), and **3i** (R = 2-F) was the most active compound in this series with MIC value of 0.024 µg/ml against *S. aureus* and *S. epidermidis.* On the other hand, screening on the Gram-negative species recorded compound **3h** bearing *meta* fluorine moiety as the most active compound.

### Anti-ureolytic activity of 3n against urease positive microorganisms

The most potent derivative in enzymatic as well as antimicrobial assay was selected to evaluate against urease positive microorganisms including standard species of *C. neoformans* (H99), and clinical isolate of *P. vulgaris*. As can be seen in Table 3, derivative **3n** significantly reduce the urease activity of tested pathogens which support the proposed therapeutic pathway to reduce urease activity against urease-positive microorganism.

**Table 3:** Antimicrobial assay and anti-ureolytic effects of **3n** against *C. neoformans* and *P. vulgaris*.

	Anti-ureolytic assay	
	IC_50_ (µg/ml) of *C. neoformans*	IC_50_ (µg/ml) of *P. vulgaris*
**3n**	5.59 ± 2.38	5.72 ± 1.31

### Molecular modeling

The docking procedure was applied based on our previous docking validity study^39^, to evaluate the interaction between newly synthesized compounds; **3a–n** against the *JB* urease active site in comparison to thiourea as a reference urease inhibitor. The top induced-fit docking (IFD) scoring pose of all compounds was analyzed inside the binding site of *JB* urease. In the binding model, all the compounds are successfully occupied in the bi-nickel active site cavity.

**Figure 4:**
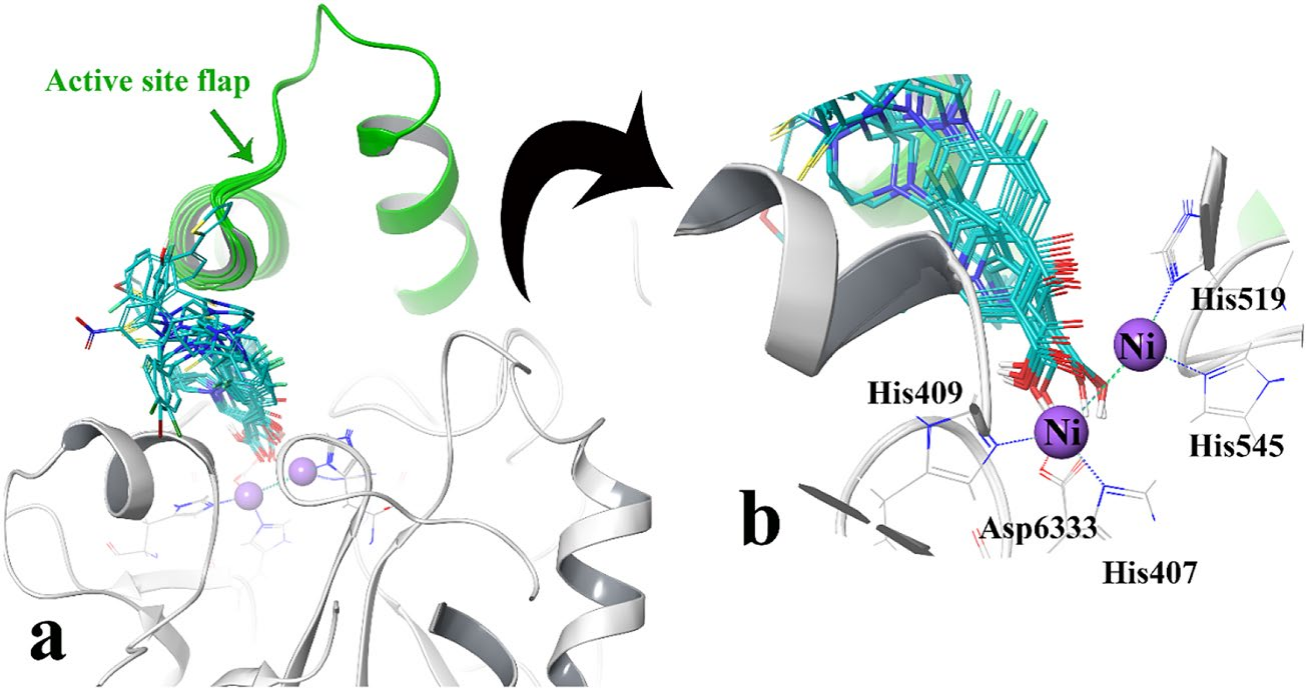
Representation of the compounds docking poses over the active site (**a**) close-up illustration of ciprofloxacin nucleuses relative to the binuclear center (**b)**, the active site flap (colored in green color).

Figure 4a shows the IFD pose of all compounds over *JB* urease. The docking results follow the cornerstone of SARs urease inhibition in which, the ciprofloxacin nucleus orients toward the two-nickel atoms through the 3-carboxylate and 4-carbonyl moiety of quinolone ring (Fig. 4b), similar to the behavior of the carbonyl oxygen in the AHA (crystallographic ligand of PDB ID: 4h9m). Also, different thioacyl moieties adopt a flexible conformation in the large hydrophobic opening of the active site flap pocket which is related to the attached substituents (Fig. 4a).

To understand the criteria for rational designing of urease inhibitors, it is necessary to uncover the structural perturbations incurred by the most potent compound (compounds **3n**) over urease and the effect of this compound on the active site environment in comparison to thiourea as the urease standard inhibitor.

**Figure 5:**
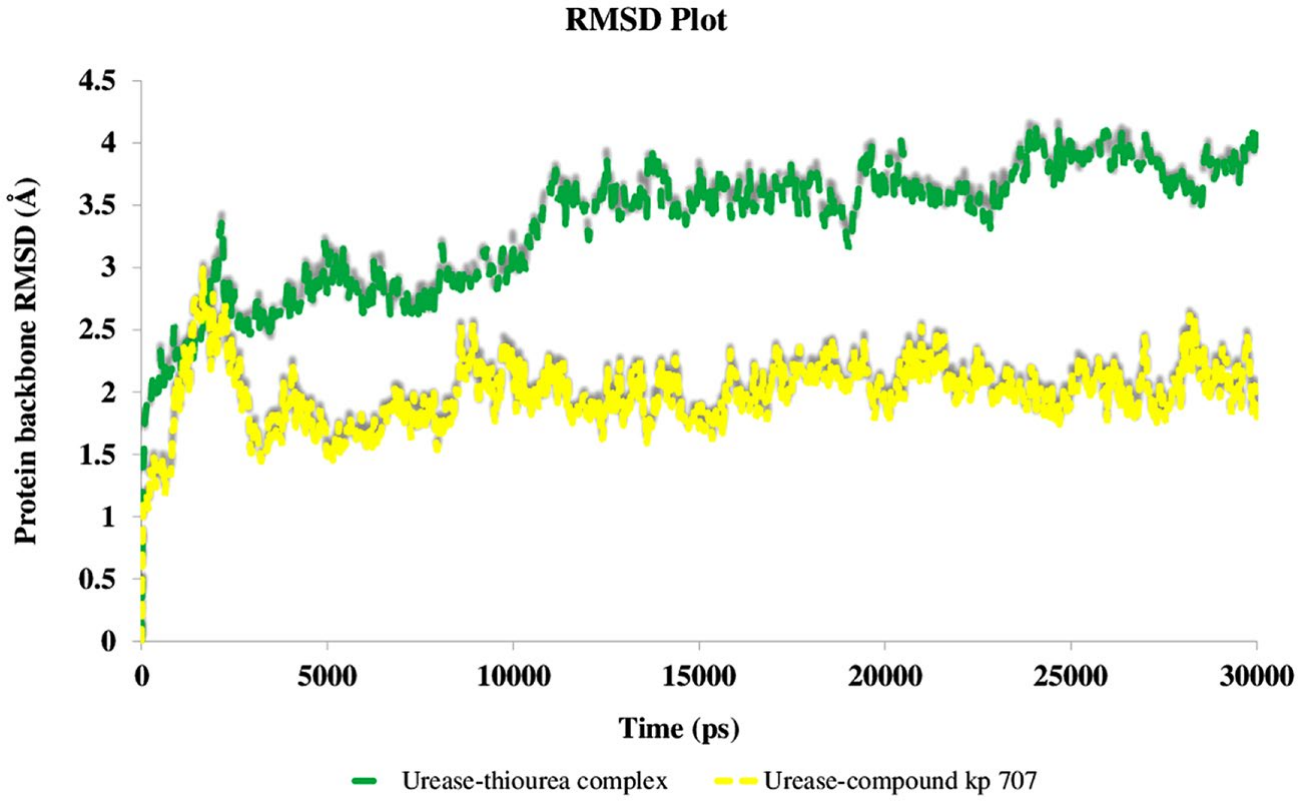
RMSD plot of the urease backbone in complexed with thiourea (in green) and compound **3n** (in yellow) over 30 ns of the MD simulation time.

To study the steadiness of the protein–ligand complex root mean square deviation (RMSD) of the protein’s backbone from its starting to terminal conformation investigated over 30 ns MD simulation. Based on the ligand-complex RMSD result it can be concluded that the engaged simulation period has been adequate to reach a balanced structure over the simulation time (Fig. 5). Therefore, the average structure at the MD equilibrium state was used to explore the structural character of the ligand–protein complexes. The green line in Fig. 5 shows urease complexed with thiourea. The RMSD simulation got overall stability after 10 ns of MD simulation time with the RMSD value at around an average of 3.80 Å while the bound-state of compound **3n** reached an equilibration state after 7 ns of MD simulation with an obviously lower RMSD value (2.2 Å) (Fig. 5, yellow line).

**Figure 6:**
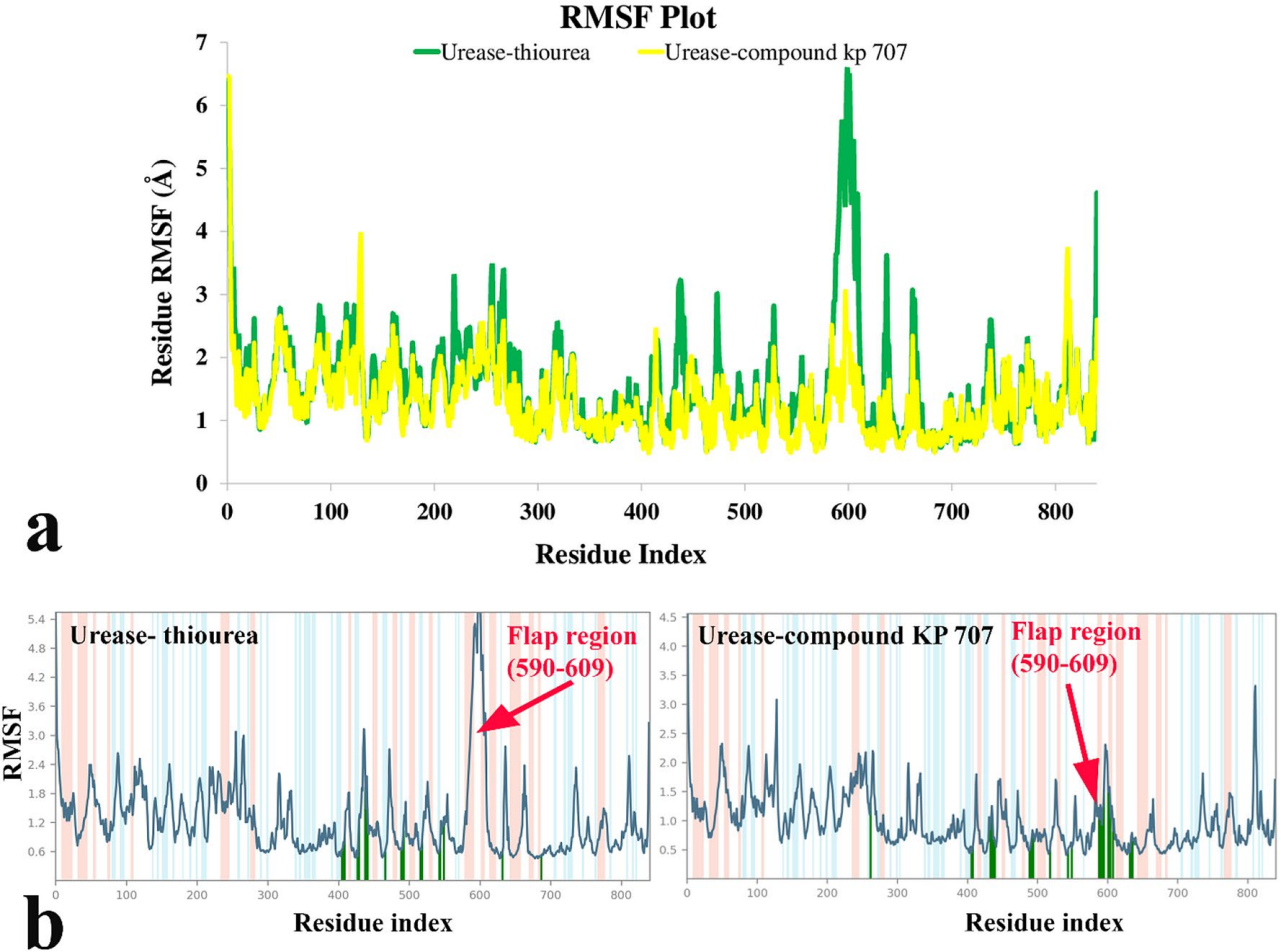
RMSF plot of the urease residue in complexed with thiourea (in green) and compound **3n** (in yellow) (**a**), individual RMSF plot regards to ligand binding location over 30 ns MD simulation time (**b**). α-helical and ß-strand regions are highlighted in light pink and blue backgrounds, respectively.

Additionally, to show the flexibility of the protein structure, the RMSF value of the protein’s residues was analyzed. Normally, the secondary structure related to α-helixes and β-sheets is more organized which show lower RMSF value while loops with loosely arranged structure show a higher one. As it is obvious from Fig. 6a the residues ranged in 590–606 with a helix-turn-helix structure, known as mobile flap region, covering the urease active site, depict significantly lower RMSF value in urease-compound **3n** complex rather than urease-thiourea complex^40^.

Furthermore, Fig. 6b shows that compound **3n** well occupied and tightly anchored the helix-turn-helix motif over the active-site cavity (vertical green line), which reduced the flexibility of the mobile flap residues (590–609) by interacting with key amino acids and results in the inhibition of urease activity.

**Figure 7:**
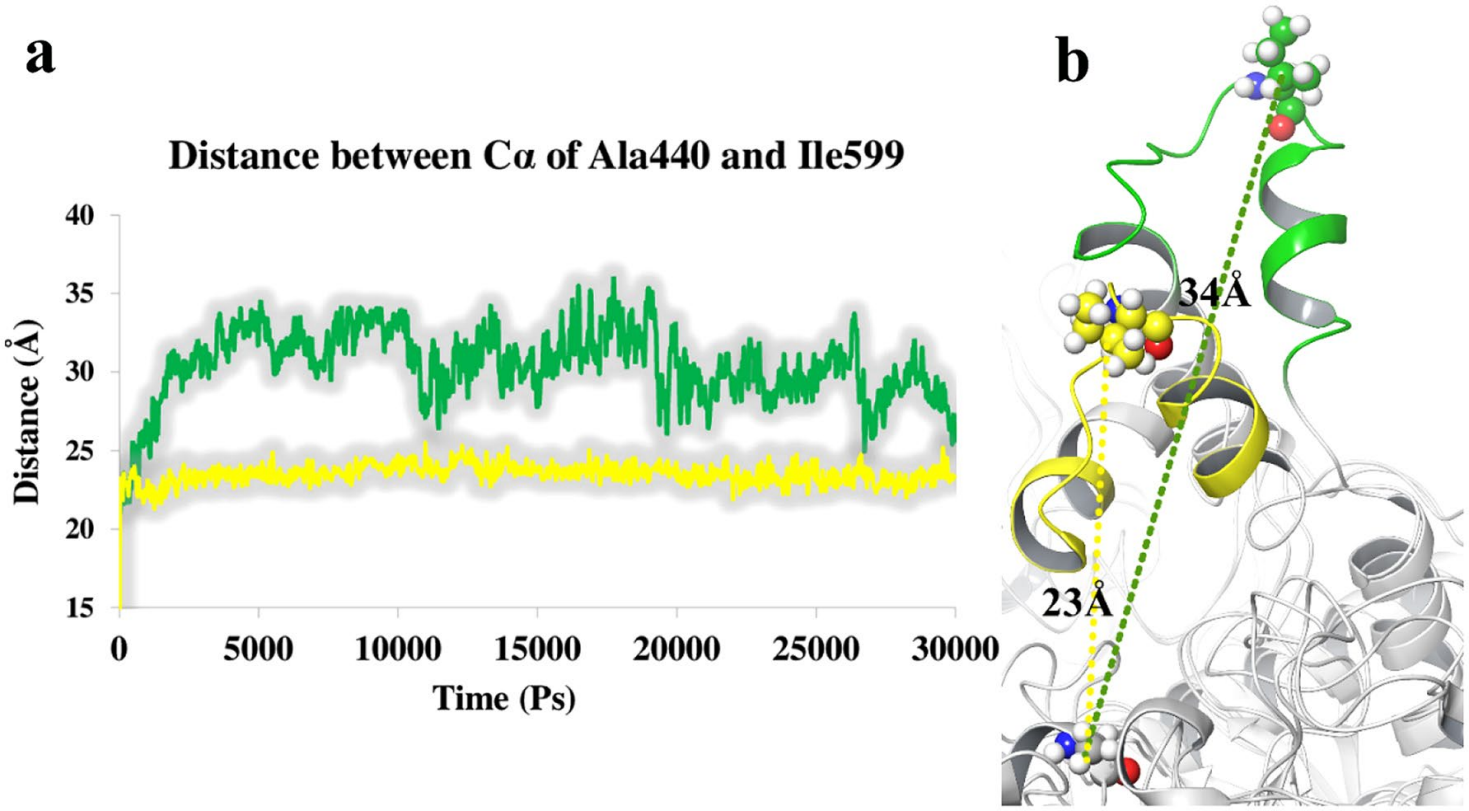
The distance between Ala440 and Ile599 urease residues when complexed with thiourea (green), and compound **3n** (yellow) during the whole MD simulation time. (**a**) Representative snapshots of MD simulations where the active site flap adopts the open (urease-thiourea complex) and closed (urease-compound **3n**) conformations which depict in green and yellow color, respectively (**b**).

In order to investigate the flexibility of the mobile flap loop during the MD simulation time, the relative length between Ile599 at the tip of the flap region and Ala440 at the root and entrance of the active site channel are analyzed and compared in urease complex with compound **3n** and thiourea, respectively. Figure 7a displays the length between Ile599 and Ala440 in urease complex with thiourea was about 34 Å corresponding to the open flap conformation, while in the case of urease complex with compound **3n** this distance swung moderately with the significantly lower value of 23 Å associated to the close flap conformation (Fig. 7b) which promote the inhibition of the ureolytic reaction through stabilizing the reaction intermediate during^41^.

**Figure 8:**
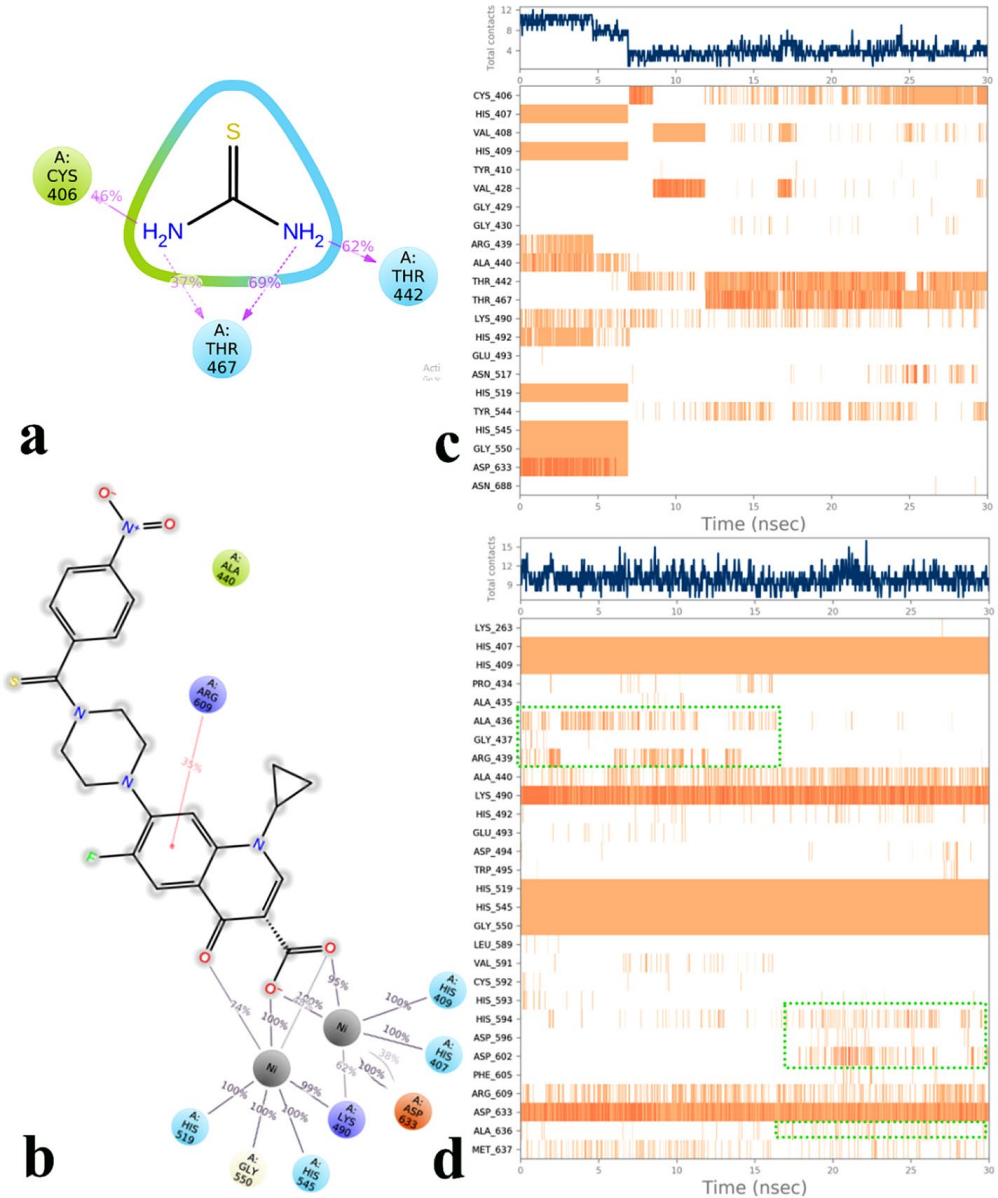
2D representation of ligand-residue interactions that occur at least 30% of simulation time at the equilibrated phase of MD simulation which include urease bound-state with thiourea (**a**) and compound **3n** (**b**). Timeline rendering of interacting residues during the whole simulation time in urease complexed with thiourea (**c**) and compound **3n** (**d**).

The molecular interactions of thiourea and compound **3n** over the binding site of urease were represented in Fig. 8. As can be seen, Fig. 8a shows thiourea formed H-bound interaction with Thr442, Thr467, and Cys405 through both of its NH_2_ groups during the equilibrated phase of MD simulation.

In addition, Fig. 8b shows the 3-carboxylate and 4-carbonyl moiety of quinolone ring of compound **3n** tightly coordinated along the metal bi-nickel center and stabilized through residues His407, His409, Lys490, His519, His545, Gly550, and Asp633 for the whole simulation time.

The interaction timeline representation depicts that thiourea provided interactions through residues His407, His409, His519, and His545 which coordinated at the bi-nuclear center of the active site for about the first quarter of MD simulation time (Fig. 8c). By progressing the simulation procedure, the mentioned interactions disappeared and some new interactions with residues Thr442, Thr467, and Cys405 emerged and produced stabilized interactions for the rest of the simulation time (Fig. 8c). The changes in interacting pattern are because of some shifting in thiourea location which results in decreasing the number of effective interactions from 8 to about 4–5 during the simulation time (Fig. 8c, the top navy plot).

Furthermore, Fig. 8d shows that for about the first half of the MD simulation time compound **3n** provided interactions with Ala436 and Arg439 in which they were disappeared and substituted by His594 and Asp602 located at the wall of the active site flap and stabilized until the end of the simulation time.

**Figure 9:**
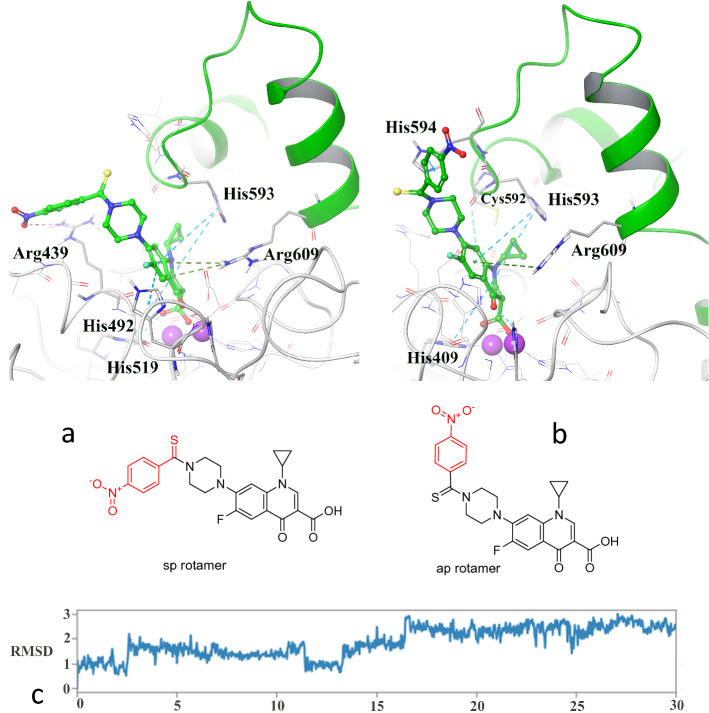
Two different configurations of 4-nitro phenyl thioamide moiety of compound **3n** from the beginning of MD to the 16.56 ns (**a**) and from the time at 16.56 ns to the end of the MD simulation time (**b**). The RMSD of compound **3n** during the whole simulation time (**c**).

To reveal the origin of this phenomenon, the trajectory file of the MD simulation to detect any changes that cause residues Ala436 and Arg439 substituted by His594, Asp602 and Ala636 over the residue interaction timeline were monitored (Fig. 8d, green dash boxes). Based on the result, it figures showed that from the beginning of the simulation time (t = 0 ns) up to 16.56 ns compound **3n** got the conformation in which the 4-nitro phenyl thioamide moiety adopted sin planar rotamer (sp rotamer) in which the thio and cyclopropyl groups oriented along the same side (Fig. 9a), while from that time the mentioned moiety instantly rotated to the anti-planar rotamer (ap rotamer) which stabilized until the end of MD simulation time (Fig. 9b). The difference in the orientation of the 4-nitro phenyl thioamide moiety results that the 4-nitro group formed electrostatic interaction with Arg439 in sp rotamer while it provided π-π stacking and electrostatic interactions with His594 and Asp602, respectively which both of them located at the sidewalls of the active site flap in ap rotamer. Also, the RMSD of compound **3n** during the whole simulation time is presented in Fig. 9c.

Moreover, in both of the conformers, the quinolone ring came up with π–π stacking and π–cation interaction with His593 and Arg609 at the root of the active site flap, respectively. It is noteworthy that His593 and His594 at one site and Arg609 at the other side of the active site flap seem to be at the strategic location because of affecting the flexibility of the mobile flap covering the active site entrance followed by inhibiting the ureolytic activity.

Finally, the MM-GBSA protocol was performed in order to uncover the impact of sp and ap rotamers on the free binding energy of compound **3n**. In this way, two sets of 100 snapshots were extracted at the time interval of 30 ps from the last 2 ns of the sp rotamer (14.56 to 16.56 ns) and ap rotamer (28 to 30 ns) which can provide scope for predicting the binding energy of compound **3n**. The calculated binding free energies (ΔG_bind_) and the individual energy components reveal that the binding free energies of urease complexed with ap rotamer is higher than sp rotamer (− 20.34 vs. − 14.65 kcal/mol, respectively) during the MD simulation time which may attribute to the more stabilizing effect of ap rotamer than sp rotamer (Table 4). Comparing other energy components show that in both of the rotamers ΔG_Coulomb_ and ΔG_vdW_ have the main contribution in providing free energy of binding which corresponds to the carboxylate ionization state and the hydrophobic character of the substitutions over the quinolone ring, respectively.

**Table 4:** Binding free energies and the individual energy terms of urease-compound **3n** complex during the first 16.56 ns (0–16.56 ns) and the rest of the simulation time (16.56–30 ns) (kcal/mol).

Energy component	Urease-compound **3n** complex sp rotamer (from t = 0 ns to t = 16.56)	Urease-compound **3n** complex ap rotamer (from t = 16.56 ns to t = 30 ns)
ΔG_bind_	− 14.65 ± 7.8	− 20.34 ± 2.9
ΔG_Coulomb_	− 19.27 ± 3.3	− 31.80 ± 5.5
ΔG_Hbond_	− 0.50 ± 0.1	− 0.70 ± 1.1
ΔG_vdW_	− 52.50 ± 4.4	− 52.46 ± 2.3
ΔG_Solv_	33.01 ± 2.7	29.27 ± 4.8

All energies are averaged over 100 snapshots and are given in kcal/mol.

### In silico prediction of pharmacokinetic properties of the synthesized compound

The main physico-chemical properties of the synthesized compounds, which represent drug-likeness, partition coefficient, solubility, and cell permeation, were calculated with the aid of the pkCSM web server (http://biosig.unimelb.edu.au/pkcsm).

Assessments of molecular weight, number of hydrogen bond donor (HBD) and hydrogen bond acceptor (HBA), calculated LogP, and defining the number of violations of Lipinski’s rule of five (ROF violations) showed that the *N-*thioacylated ciprofloxacin scaffold meets the Lipinski drug-likeness criteria.

Relay on the impact of solubility and permeability on gastrointestinal absorption^42^, some relevant physico-chemical parameters like predicted aqueous solubility (Log *S*), the predicted permeability (LogCaco-2), and the predicted % human intestinal absorption (% HIA) were computed for newly synthesized compounds **3a–n** and hydroxyurea (Table 5). Based on the obtained value, all compounds showed favorable solubility and exhibited high HIA in which their value is higher than 30%.

**Table 5:** Physico-chemical properties of compounds **3a–n**.

Compound	Mw	HBD	HBA	LogP	ROF^a^	Log S^b^	Log Caco-2^c^	%HIA^d^
**3a**	453.54	1	5	3.19	0	− 4.53	1.09	92.22
**3b**	483.56	1	6	3.19	0	− 4.20	1.38	94.53
**3c**	467.57	1	5	3.49	0	− 4.13	1.32	94.25
**3d**	471.52	1	5	3.32	0	− 4.66	1.21	92.46
**3e**	487.98	1	5	3.8	0	− 5.03	1.19	91.48
**3f**	483.56	1	6	3.19	0	− 4.21	1.36	94.61
**3g**	498.53	1	7	3.09	0	− 4.97	0.73	71.79
**3h**	471.52	1	5	3.32	0	− 4.66	1.21	92.31
**3i**	471.52	1	5	3.32	0	− 4.56	1.21	92.52
**3j**	469.53	2	6	2.89	0	− 4.18	1.23	91.73
**3k**	545.63	1	6	4.9	1	− 5.64	1.00	92
**3l**	532.43	1	5	3.9	1	− 5.07	1.18	91.23
**3m**	487.98	1	5	3.8	0	− 5	1.18	91.30
**3n**	498.53	1	7	3	0	− 4.98	0.7	71
Hydroxyurea	76.05	3	2	− 0.9	0	0.7	0.4	73

^a^Number of violations of Lipinski’s rule of five.

^b^Predicted aqueous solubility in mol/l (− 6.5 to 0.5) (QPlogS > − 5.7).

^c^Predicted Caco-2 cell permeability of a given compound is given as the log P_app_ in 10^−6^ cm/s (high Caco-2 permeability has LogCaco-2 > 0.9).

^d^Percent of human intestinal absorption, (< 30% is poor and > 30% is high).

## Conclusion

In conclusion, a new series of *N-*thioacylated ciprofloxacin **3a–n** were synthesized based on a facile Willgerodt-Kindler-type reaction under catalyst‐free conditions at a mild temperature. Besides, the in vitro antibacterial activity of the compounds was examined on two Gram-negative bacteria, *E. coli* and *P. aeruginosa*, and two Gram-positive bacteria, *S. aureus*, and *S. epidermidis.* A considerable number of compounds displayed better activity against *S. epidermidis* in comparison with the parent drug ciprofloxacin*.* Some of the compounds exhibited similar activity to ciprofloxacin against *S. aureus.* Among the derivatives, compound **3g** displayed perfect activity against *S. aureus, S. epidermidis,* and *E. coli*. This compound’s activity against *S. epidermidis* was twofold higher than that of ciprofloxacin, and its activity against *S. aureus* was similar to the value for the parent drug. In addition, compound **3h** showed similar activity to ciprofloxacin against *P. aeruginosa* and *E. coli*. Furthermore, these compounds showed excellent inhibitory activity against *JB* urease enzyme in comparison with the standard inhibitors (hydroxyurea and thiourea). The obtained results revealed that almost all the title compounds **3a–n** were more potent than standard urease inhibitors. Moreover, the compounds **3d,** **3e, 3g**, **3h**, **3l**, and especially **3n** were highly potent with IC_50_ values less than 10 µM. IFD investigation and MD simulations showed that compound **3n** exhibited pronounced interaction with essential urease active site and mobile flap residues through the quinolone ring by coordinating toward the metal bi-nickel complex and the essential residues at the active site flap-like His593, His594, and Arg609, respectively. In addition, the results uncover the prominent ap rotamer of the 4-nitro phenyl thioamide derivative, which is the energetically favorable conformation rather than sn rotamer. The compatibility investigation of the compounds for both antibacterial and urease inhibitory activities revealed that the most pronounced compounds for the mentioned assays are **3g** and **3h** compounds, in which fluoro and nitro substituents were located at *meta* position, respectively. **3n** also recorded IC_50_ values of 5.59 ± 2.38 and 5.72 ± 1.312 µg/ml to inhibit urease enzyme against *C. neoformans* and *P.vulgaris* in the ureolytic assay.

Without a doubt, the results of these structures can be construed as a lead compound for further investigations.

## Materials and methods

Ciprofloxacin and sulfur, and dimethyl sulfoxide were purchased from Sigma-Aldrich, and aldehydes were obtained from Merck. All chemicals and solvents employed in this research were of analytical grade. Melting points were also determined on a Kofler hot stage apparatus and reported uncorrected. ^1^H and ^13^C NMR spectra were also recorded on a Bruker FT-300, using TMS as an internal standard. IR spectra were taken by a Nicolet Magna FTIR 550 spectrophotometer (KBr disks). Elemental analysis was additionally performed on an Elementar Analysen system GmbH VarioEL CHNS mode.

### General procedure for the synthesis of N-thioacylated ciprofloxacin derivatives 3a–n (Fig. S1-14)

A mixture of ciprofloxacin **1** (1 mmol), aromatic aldehydes **2a–n** (1 mmol), and sulfur (4.0 mmol) in DMSO (3 mL) was stirred at 50 °C for 5–8 h at the closed condition. Then, the mixture was poured into the cold water, and the pure final derivatives **3a–n** were filtered off. Recrystallization in ethanol was later on used to give pure target products.

#### 1-Cyclopropyl-6-fluoro-4-oxo-7-(4-(phenylcarbonothioyl)piperazin-1-yl)-1,4 dihydroquinoline-3-carboxylic acid 3a

Yield 83%; yellow solid; mp > 250 °C. IR (KBr): 3436, 3029, 1716, 1178 cm^−1^. ^1^H NMR (301 MHz, DMSO-*d*_6_) δ 8.66 (s, 1H), 7.91 (d, *J* = 13.2 Hz, 1H), 7.59 (d, *J* = 7.4 Hz, 1H), 7.53–7.28 (m, 3H), 4.55 (t, *J* = 5.2 Hz, 2H), 3.82 (h, *J* = 5.1 Hz, 3H), 3.64 (t, *J* = 5.3 Hz, 2H), 3.30–3.16 (m, 2H), 1.44–1.29 (m, 2H), 1.28–1.12 (m, 2H). ^13^C NMR (75 MHz, DMSO-*d*_6_) *δ* 199.56, 176.78 (^4^*J*_C–F_ = 2.25 Hz), 166.35, 154.87 (^1^*J*_C–F_ = 247.5 Hz), 148.51 (^4^*J*_C–F_ = 3.75 Hz), 144.841 (^3^*J*_C–F_ = 9.75 Hz), 142.94, 139.56, 129.13, 128.78, 126.40, 119.29 (^3^*J*_C–F_ = 7.5 Hz), 111.65 (^2^*J*_C–F_ = 21 Hz), 107.21, 106.98 (^4^*J*_C–F_ = 2.25 Hz), 51.48, 48.91, 36.40, 8.10 ppm. Anal. calcd. For C_24_H_22_FN_3_O_3_S: C, 63.84; H, 4.91; N, 9.31. Found: C, 63.94; H, 4.99; N, 9.21.

#### 1-Cyclopropyl-6-fluoro-7-(4-(3-methoxyphenylcarbonothioyl)piperazin-1-yl)-4-oxo-1,4-dihydroquinoline-3-carboxylic acid 3b

Yield 87%; yellow solid; mp > 250 °C. IR (KBr): 3436, 3016, 1717, 1181 cm^−1^. ^1^H NMR (301 MHz, DMSO-*d*_6_) δ 8.63 (s, 1H), 7.86 (d, *J* = 13.2 Hz, 1H), 7.56 (d, *J* = 7.4 Hz, 1H), 7.37 (d, *J* = 8.6 Hz, 1H), 6.98 (d, *J* = 8.7 Hz, 1H), 4.52 (t, *J* = 4.7 Hz, 2H), 3.89 (d, *J* = 4.4 Hz, 2H), 3.82 (s, 3H), 3.63 (d, *J* = 5.6 Hz, 2H), 3.49–3.38 (m, 3H), 3.22 (t, *J* = 5.0 Hz, 1H), 1.35 (d, *J* = 6.9 Hz, 2H), 1.18 (d, *J* = 4.6 Hz, 2H). ^13^C NMR (75 MHz, DMSO-*d*_*6*_) *δ* 199.77, 176.71 (^4^*J*_C–F_ = 2.25 Hz), 166.32, 160.20, 154.82 (^1^*J*_C–F_ = 247.5 Hz), 148.36, 144.84 (^3^*J*_C–F_ = 10.5 Hz), 139.52, 135.26, 132.25, 128.69, 119.17 (^3^*J*_C–F_ = 7.5 Hz), 114.96, 113.91, 111.59 (^2^*J*_C–F_ = 21 Hz), 107.18, 106.83 (^4^*J*_C–F_ = 3 Hz), 55.80, 51.70, 49.35, 36.37, 8.09 ppm. Anal. calcd. For C_25_H_24_FN_3_O_4_S: C, 62.36; H, 5.02; N, 8.73. Found: C, 62.56; H, 5.22; N, 8.59.

#### 1-Cyclopropyl-6-fluoro-7-(4-(4-methylphenylcarbonothioyl)piperazin-1-yl)-4-oxo-1,4-dihydroquinoline-3-carboxylic acid 3c

Yield 90%; yellow solid; mp > 250 °C. IR (KBr): 3441, 3023, 1715, 1183 cm^−1^. ^1^H NMR (300 MHz, DMSO-*d*_6_) δ 15.19 (s, 1H), 8.67 (s, 1H), 7.93 (d, *J* = 13.2 Hz, 1H), 7.59 (d, *J* = 7.5 Hz, 1H), 7.36–7.14 (m, 4H), 4.54 (t, *J* = 5.0 Hz, 2H), 3.83 (dd, *J* = 7.2, 3.8 Hz, 3H), 3.62 (t, *J* = 5.3 Hz, 2H), 3.43 (t, *J* = 5.1 Hz, 2H), 2.36 (s, 3H), 1.34 (dd, *J* = 7.5, 5.5 Hz, 2H), 1.25–1.12 (m, 2H). ^13^C NMR (75 MHz, DMSO-*d*_6_) *δ* 199.86, 176.82 (^4^*J*_C–F_ = 3 Hz), 166.37, 154.89 (^1^*J*_C–F_ = 247.5 Hz), 148.51, 144.87 (^3^*J*_C–F_ = 9.75 Hz), 140.21, 139.59, 138.86, 129.20, 126.60, 119.32 (^3^*J*_C–F_ = 8.25 Hz), 111.67 (^2^*J*_C–F_ = 22.5 Hz), 107.22, 107.01 (^4^*J*_C–F_ = 3 Hz), 51.53, 49.04, 36.40, 21.28, 8.10 ppm. Anal. calcd. For C_25_H_24_FN_3_O_3_S: C, 64.50; H, 5.20; N, 9.03. Found: C, 62.66; H, 5.08; N, 9.23.

#### 1-Cyclopropyl-6-fluoro-7-(4-(4-fluorophenylcarbonothioyl)piperazin-1-yl)-4-oxo-1,4-dihydroquinoline-3-carboxylic acid 3d

Yield 92%; yellow solid; mp > 250 °C. IR (KBr): 3454, 3032, 1719, 1190 cm^−1^. ^1^H NMR (301 MHz, DMSO-*d*_6_) δ 15.17 (s, 1H), 8.66 (s, 1H), 7.91 (d, *J* = 13.2 Hz, 1H), 7.64–7.42 (m, 2H), 7.34–7.13 (m, 3H), 4.53 (t, *J* = 5.2 Hz, 2H), 3.81 (dd, *J* = 6.9, 3.6 Hz, 3H), 3.68–3.59 (m, 2H), 3.46 (t, *J* = 5.2 Hz, 2H), 1.34 (dd, *J* = 7.5, 5.5 Hz, 2H), 1.25–1.14 (m, 2H). ^13^C NMR (75 MHz, DMSO-*d*_6_) *δ* 197.36, 176.77 (^4^*J*_C–F_ = 3 Hz), 166.34, 163.73 (^1^*J*_C–F_ = 243 Hz), 154.84 (^1^*J*_C–F_ = 246.75 Hz), 148.45, 144.93 (^3^*J*_C–F_ = 7.5 Hz), 144.80 (^3^*J*_C–F_ = 12 Hz), 139.56, 131.09 (^3^*J*_C–F_ = 8.25 Hz), 122.49 (^4^*J*_C–F_ = 3 Hz), 119.27 (^3^*J*_C–F_ = 7.5 Hz), 116.02 (^2^*J*_C–F_ = 21 Hz), 113.62 (^2^*J*_C–F_ = 22.5 Hz), 111.66 (^2^*J*_C–F_ = 22.5 Hz), 107.21, 106.91 (^4^*J*_C–F_ = 3 Hz), 51.53, 48.85, 36.39, 8.10 ppm. Anal. calcd. For C_24_H_21_F_2_N_3_O_3_S: C, 61.40; H, 4.51; N, 8.95. Found: C, 61.26; H, 4.36; N, 9.03.

#### 7-(4-(4-Chlorophenylcarbonothioyl)piperazin-1-yl)-1-cyclopropyl-6-fluoro-4-oxo-1,4-dihydroquinoline-3-carboxylic acid 3e

Yield 91%; yellow solid; mp > 250 °C. IR (KBr): 3433, 3019, 1720, 1188 cm^−1^. ^1^H NMR (301 MHz, DMSO-*d*_6_) δ 15.17 (s, 1H), 8.66 (s, 1H), 7.92 (d, *J* = 13.2 Hz, 1H), 7.58 (d, *J* = 7.5 Hz, 1H), 7.55–7.48 (m, 2H), 7.46–7.38 (m, 2H), 4.53 (t, *J* = 5.1 Hz, 2H), 3.83–6.78 (m, 3H), 3.64 (t, *J* = 5.3 Hz, 2H), 3.46 (t, *J* = 5.4 Hz, 2H), 1.34 (dd, *J* = 7.5, 5.5 Hz, 2H), 1.19 (dt, *J* = 7.5, 5.1 Hz, 2H). ^13^C NMR (75 MHz, DMSO-*d*_6_) δ 198.00, 176.79 (^4^*J*_C–F_ = 2.25 Hz), 166.34, 154.85 (^1^*J*_C–F_ = 246.75 Hz), 148.49, 144.80 (^3^*J*_C–F_ = 10.5 Hz), 141.65, 139.57, 133.79, 128.83, 128.38, 119.29 (^3^*J*_C–F_ = 7.5 Hz), 111.68 (^2^*J*_C–F_ = 22.5 Hz), 107.23, 106.94 (^4^*J*_C–F_ = 3.75 Hz), 106.89, 51.58, 48.98, 36.39, 8.10 ppm. Anal. calcd. For C_24_H_21_ClFN_3_O_3_S: C, 59.32; H, 4.36; N, 8.65. Found: C, 59.57; H, 4.43; N, 8.47.

#### 1-Cyclopropyl-6-fluoro-7-(4-(4-methoxyphenylcarbonothioyl)piperazin-1-yl)-4-oxo-1,4-dihydroquinoline-3-carboxylic acid 3f

Yield 91%; yellow solid; mp > 250 °C. IR (KBr): 3438, 3019, 1715, 1176 cm^−1^. ^1^H NMR (301 MHz, DMSO-*d*_6_) δ 15.17 (s, 1H), 8.65 (s, 1H), 7.90 (d, *J* = 13.2 Hz, 1H), 7.58 (d, *J* = 6.6 Hz, 1H), 7.37 (dd, *J* = 8.6, 2.1 Hz, 2H), 6.99 (dd, *J* = 8.7, 2.2 Hz, 2H), 4.52 (s, 2H), 3.88–3.83 (m, 3H), 3.72 (s, 3H), 3.65 (s, 2H), 3.47 (s, 2H), 1.36 (d, *J* = 5.3 Hz, 2H), 1.19 (s, 2H). ^13^C NMR (75 MHz, DMSO-*d*_6_) δ 199.15, 176.76 (^4^*J*_C–F_ = 3 Hz), 166.34, 154.83 (^1^*J*_C–F_ = 247.5 Hz), 153.04, 148.44, 144.85 (^3^*J*_C–F_ = 10.5 Hz), 139.56, 138.31, 138.07, 119.18 (^4^*J*_C–F_ = 7.5 Hz), 111.65 (^2^*J*_C–F_ = 22.5 Hz), 107.19, (^4^*J*_C–F_ = 3.75 Hz), 104.19, 56.55, 51.65, 49.03, 36.39, 8.10 ppm. Anal. calcd. For C_25_H_24_FN_3_O_4_S: C, 62.36; H, 5.02; N, 8.73. Found: C, 62.49; H, 5.14; N, 8.59.

#### 1-Cyclopropyl-6-fluoro-7-(4-(3-nitrophenylcarbonothioyl)piperazin-1-yl)-4-oxo-1,4-dihydroquinoline-3-carboxylic acid 3g

Yield 91%; orange solid; mp > 250 °C. IR (KBr): 3452, 3029, 1724, 1187 cm^−1^. ^1^H NMR (301 MHz, DMSO-*d*_6_) δ 15.16 (s, 1H), 8.66 (s, 1H), 8.30–8.19 (m, 2H), 7.97–7.68 (m, 3H), 7.58 (d, *J* = 7.4 Hz, 1H), 4.56 (t, *J* = 4.9 Hz, 2H), 3.84 (dd, *J* = 6.9, 3.6 Hz, 3H), 3.68 (t, *J* = 5.3 Hz, 2H), 3.48 (d, *J* = 5.2 Hz, 2H), 1.35 (d, *J* = 6.8 Hz, 2H), 1.20 (d, *J* = 3.7 Hz, 2H). ^13^C NMR (75 MHz, DMSO-*d*_6_) *δ* 196.05, 176.76 (^4^*J*_C–F_ = 3 Hz), 166.32, 154.82 (^1^*J*_C–F_ = 246.75 Hz), 148.46, 148.02, 144.76 (^3^*J*_C–F_ = 9.75 Hz), 144.05, 139.55, 135.37, 132.64, 130.58, 123.75, 121.31, 119.24 (^3^*J*_C–F_ = 8.25 Hz), 111.67 (^2^*J*_C–F_ = 22.5 Hz), 107.21, 106.84 (^4^*J*_C–F_ = 3 Hz), 51.70, 48.97, 36.37, 8.11 ppm. Anal. calcd. For C_24_H_21_FN_4_O_5_S: C, 58.06; H, 4.26; N, 11.28. Found: C, 58.29; H, 5.34; N, 11.09.

#### 1-Cyclopropyl-6-fluoro-7-(4-(3-fluorophenylcarbonothioyl)piperazin-1-yl)-4-oxo-1,4-dihydroquinoline-3-carboxylic acid 3h

Yield 84%; yellow solid; mp > 250 °C. IR (KBr): 3446, 3032, 1724, 1189 cm^−1^. ^1^H NMR (301 MHz, DMSO-*d*_6_) δ 15.01 (s, 1H), 8.61 (s, 1H), 7.83 (d, *J* = 13.2 Hz, 1H), 7.73–7.37 (m, 2H), 7.37–7.05 (m, 3H), 4.53 (t, *J* = 5.1 Hz, 2H), 3.80 (dq, *J* = 8.8, 4.5, 4.1 Hz, 3H), 3.64 (d, *J* = 5.3 Hz, 2H), 3.46 (d, *J* = 5.4 Hz, 2H), 1.44–1.29 (m, 2H), 1.18 (p, *J* = 5.8, 5.2 Hz, 2H). ^13^C NMR (75 MHz, DMSO-*d*_6_) *δ* 197.40 (^4^*J*_C–F_ = 1.5 Hz), 176.65 (^4^*J*_C–F_ = 2.25 Hz), 166.28, 163.73 (^1^*J*_C–F_ = 243 Hz), 154.78 (^1^*J*_C–F_ = 247.5 Hz), 148.29, 144.92 (^3^*J*_C–F_ = 7.5 Hz), 144.75 (^3^*J*_C–F_ = 10.5 Hz), 139.48, 131.08, (^3^*J*_C–F_ = 8.25 Hz), 122.48 (^4^*J*_C–F_ = 2.25 Hz), 119.15 (^3^*J*_C–F_ = 7.5 Hz), 116.01 (^2^*J*_C–F_ = 20.25 Hz), 113.63 (^2^*J*_C–F_ = 22.5 Hz), 111.57 (^2^*J*_C–F_ = 23.25 Hz), 107.16, 106.77 (^4^*J*_C–F_ = 3.75 Hz), 51.53, 48.87, 36.35, 8.09 ppm. Anal. calcd. For C_24_H_21_F_2_N_3_O_3_S: C, 61.40; H, 4.51; N, 8.95. Found: C, 61.31; H, 4.34; N, 9.09.

#### 1-Cyclopropyl-6-fluoro-7-(4-(2-fluorophenylcarbonothioyl)piperazin-1-yl)-4-oxo-1,4-dihydroquinoline-3-carboxylic acid 3i

Yield 81%; yellow solid; mp > 250 °C. IR (KBr): 3450, 3037, 1725, 1190 cm^−1^. ^1^H NMR (300 MHz, DMSO-*d*_6_) *δ* 15.11 (s, 1H), 8.63 (s, 1H), 8.17 (dt, *J* = 8.3, 0.8 Hz, 1H), 8.08 (dt, *J* = 8.3, 1.0 Hz, 1H), 7.93–7.81 (m, 1H), 7.62–7.36 (m, 3H), 4.56 (t, *J* = 5.0 Hz, 2H), 4.00–3.53 (m, 7H), 1.55–0.79 (m, 4H). ^13^C NMR (75 MHz, DMSO-*d*_6_) *δ* 192.36 (^4^*J*_C–F_ = 3 Hz),176.7448 (^4^*J*_C–F_ = 2.25 Hz), 166.30, 163.78 (^1^*J*_C–F_ = 242.25 Hz), 154.85 (^1^*J*_C–F_ = 246 Hz), 153.52, 148.41, 148.32, 145.22 (^3^*J*_C–F_ = 10.5 Hz), 140.94, 139.50, (^4^*J*_C–F_ = 3.75 Hz), 133.77, 127.77, 126.95 (^4^*J*_C–F_ = 2.25 Hz), 125.73, 119.33 (^3^*J*_C–F_ = 7.5 Hz), 111.64 (^2^*J*_C–F_ = 23.25 Hz), 111.57 (^2^*J*_C–F_ = 22.5 Hz), 107.22, 107.15 (^4^*J*_C–F_ = 2.25 Hz), 51.71, 49.66, 36.36, 8.09 ppm. Anal. calcd. For C_24_H_21_F_2_N_3_O_3_S: C, 61.40; H, 4.51; N, 8.95. Found: C, 61.35; H, 4.38; N, 9.02.

#### 1-Cyclopropyl-6-fluoro-7-(4-(3-hydroxyphenylcarbonothioyl)piperazin-1-yl)-4-oxo-1,4-dihydroquinoline-3-carboxylic acid 3j

Yield 84%; yellow solid; mp > 250 °C. IR (KBr): 3446, 3019, 1715, 1176 cm^−1^. ^1^H NMR (301 MHz, DMSO-*d*_6_) δ 15.15 (s, 1H), 9.75 (s, 1H), 8.64 (s, 1H), 7.88 (d, *J* = 13.1 Hz, 1H), 7.58 (d, *J* = 7.4 Hz, 1H), 7.28 (t, *J* = 7.8 Hz, 1H), 6.94–6.74 (m, 2H), 4.52 (t, *J* = 5.1 Hz, 2H), 4.13–3.41 (m, 7H), 1.42–1.26 (m, 2H), 1.20 (dd, *J* = 6.1, 3.7 Hz, 2H). ^13^C NMR (75 MHz, DMSO-*d*_6_) *δ* 193.59, 176.76 (^4^*J*_C–F_ = 2.25 Hz), 169.52, 166.32, 157.83, 155.03 (^1^*J*_C–F_ = 247.5 Hz), 148.45, 148.40, 145.38 (^3^*J*_C–F_ = 9.75 Hz), 139.54, 137.30, 130.07, 119.34 (^3^*J*_C–F_ = 8.25 Hz), 117.87, 117.07, 114.31, 111.57 (^2^*J*_C–F_ = 22.5 Hz), 107.20, 107.17 (^4^*J*_C–F_ = 3 Hz), 51.76, 49.92, 36.36, 8.07 ppm. Anal. calcd. For C_24_H_22_FN_3_O_4_S: C, 61.66; H, 4.74; N, 8.99. Found: C, 61.71; H, 4.83; N, 8.86.

#### 1-Cyclopropyl-6-fluoro-4-oxo-7-(4-(3-phenoxyphenylcarbonothioyl)piperazin-1-yl)-1,4-dihydroquinoline-3-carboxylic acid 3k

Yield 78%; yellow solid; mp > 250 °C. IR (KBr): 3441, 3019, 1716, 1174 cm^−1^. ^1^H NMR (301 MHz, DMSO-*d*_6_) δ 15.16 (s, 1H), 8.64 (s, 1H), 7.89 (d, *J* = 13.2 Hz, 1H), 7.56 (d, *J* = 7.4 Hz, 1H), 7.45 (ddd, *J* = 10.9, 6.1, 2.9 Hz, 3H), 7.26–6.97 (m, 6H), 4.51 (t, *J* = 5.1 Hz, 2H), 3.81 (dq, *J* = 15.0, 3.5 Hz, 3H), 3.62 (t, *J* = 5.2 Hz, 2H), 3.45 (d, *J* = 5.5 Hz, 2H), 1.44–1.28 (m, 2H), 1.25–1.12 (m, 2H). ^13^C NMR (75 MHz, DMSO-*d*_6_) δ 198.24, 176.74 (^4^*J*_C–F_ = 2.25 Hz), 166.33, 157.05, 156.53, 154.84 (^1^*J*_C–F_ = 247.5 Hz), 148.40, 144.80 (^3^*J*_C–F_ = 10.5 Hz), 144.56, 139.53, 130.65, 130.61, 124.39, 121.19, 119.80, 119.47, 119.23 (^3^*J*_C–F_ = 7.5 Hz), 118.83, 116.30, 111.63 (^2^*J*_C–F_ = 22.5 Hz), 107.20, 106.89 (^4^*J*_C–F_ = 3.75 Hz), 51.54, 48.84, 36.37, 8.11 ppm. Anal. calcd. For C_30_H_26_FN_3_O_4_S: C, 66.28; H, 4.82; N, 7.73. Found: C, 66.55; H, 4.96; N, 7.68.

#### 7-(4-(4-Bromophenylcarbonothioyl)piperazin-1-yl)-1-cyclopropyl-6-fluoro-4-oxo-1,4-dihydroquinoline-3-carboxylic acid 3l

Yield 91%; yellow solid; mp > 250 °C. IR (KBr): 3439, 3020, 1716, 1180 cm^−1^. ^1^H NMR (301 MHz, DMSO-*d*_6_) δ 15.18 (s, 1H), 8.68 (s, 1H), 7.93 (d, *J* = 13.0 Hz, 1H), 7.69–7.46 (m, 3H), 7.34 (t, *J* = 8.7 Hz, 2H), 4.45 (t, *J* = 5.1 Hz, 1H), 3.68 (d, *J* = 83.1 Hz, 7H), 1.53–1.07 (m, 4H). ^13^C NMR (76 MHz, DMSO-*d*_6_) *δ* 194.95, 176.86 (^4^*J*_C–F_ = 2.25 Hz), 166.40, 155.08 (^1^*J*_C–F_ = 246.75 Hz), 148.56, 145.41 (^3^*J*_C–F_ = 10.5 Hz), 139.61, 132.44, 132.40, 130.28, 130.16, 119.43 (^3^*J*_C–F_ = 7.5 Hz), 116.09, 115.80, 111.66 (^2^*J*_C–F_ = 22.5 Hz), 107.24, 106.98 (^4^*J*_C–F_ = 2.25 Hz), 51.49, 49.83, 36.39, 8.09 ppm. Anal. calcd. For C_24_H_21_BrFN_3_O_3_S: C, 54.35; H, 3.99; N, 7.92. Found: C, 54.47; H, 4.06; N, 7.78.

#### 7-(4-(3-Chlorophenylcarbonothioyl)piperazin-1-yl)-1-cyclopropyl-6-fluoro-4-oxo-1,4-dihydroquinoline-3-carboxylic acid 3m

Yield 82%; yellow solid; mp > 250 °C. IR (KBr): 3435, 3016, 1712, 1176 cm^−1^. ^1^H NMR (301 MHz, DMSO-*d*_6_) δ 15.08 (s, 1H), 8.61 (s, 1H), 7.81 (d, *J* = 13.2 Hz, 1H), 7.64–7.40 (m, 4H), 7.33 (td, *J* = 4.1, 2.0 Hz, 1H), 4.52 (t, *J* = 5.1 Hz, 2H), 4.00–3.41 (m, 7H), 1.39–1.32 (m, 2H), 1.21–1.15 (m, 2H). ^13^C NMR (76 MHz, DMSO-*d*_6_) *δ* 197.16, 176.66 (^4^*J*_C–F_ = 2.25 Hz), 166.25, 154.96 (^1^*J*_C–F_ = 246 Hz), 148.28, 145.28 (^3^*J*_C–F_ = 9.75 Hz), 144.66, 139.46, 138.16, 133.76, 130.97, 130.08, 127.34, 126.11, 119.24 (^3^*J*_C–F_ = 7.5 Hz), 111.52 (^2^*J*_C–F_ = 22.5 Hz), 107.18, 107.01 (^4^*J*_C–F_ = 3 Hz), 51.58, 48.88, 36.33, 8.08 ppm. Anal. calcd. For C_24_H_21_ClFN_3_O_3_S: C, 59.32; H, 4.36; N, 8.65. Found: C, 59.54; H, 4.47; N, 8.52.

#### 1-Cyclopropyl-6-fluoro-7-(4-(4-nitrophenylcarbonothioyl)piperazin-1-yl)-4-oxo-1,4-dihydroquinoline-3-carboxylic acid 3n

Yield 94%; orange solid; mp > 250 °C. IR (KBr): 3451, 3033, 1724, 1193 cm^−1^. ^1^H NMR (400 MHz, DMSO-*d*_6_) δ 8.66 (s, 1H), 8.29 (d, *J* = 8.4 Hz, 2H), 7.92 (d, *J* = 8.4 Hz Hz, 2H), 7.77 (d, *J* = 8.2 Hz, 1H), 7.62 (d, *J* = 8.3 Hz, 1H), 4.54 (t, *J* = 4.7 Hz, 1H), 3.96–3.72 (m, 3H), 3.66 (s, 2H), 3.40–3.36 (m, 2H), 1.33 (d, *J* = 6.5 Hz, 2H), 1.26–1.10 (m, 2H). ^13^C NMR (100 MHz, DMSO-*d*_6_) *δ* 196.29, 176.80 (^4^*J*_C–F_ = 1.5 Hz), 166.36, 154.45 (^1^*J*_C–F_ = 248 Hz), 148.54, 147.39, 144.87 (^3^*J*_C–F_ = 11 Hz), 139.55, 128.92, 127.51, 124.33, 119.52 (^3^*J*_C–F_ = 10 Hz), 111.67 (^2^*J*_C–F_ = 23 Hz), 107.23 (^4^*J*_C–F_ = 3 Hz), 107.00, 51.55, 48.63, 36.41, 8.08 ppm. Anal. calcd. For C_24_H_21_FN_4_O_5_S: C, 58.06; H, 4.26; N, 11.28. Found: C, 58.24; H, 5.37; N, 11.14.

### In vitro urease inhibitory assay

All chemicals and *jack bean* urease (JBU; EC 3.5.1.5) were purchased from Sigma-Aldrich. The urease inhibitory activity of *N-*thioacylated ciprofloxacin derivatives **3a–n** was determined by Berthelot colorimetric method. The ammonia (NH_3_) produced by the urease enzyme along with indicator solutions including hypochlorite (OCl^−^) and phenol make a blue-colored indophenol complex and the absorbance was measured at 625 nm by a Synergy H1 Hybrid multimode microplate reader (BioTek, Winooski, VT, USA).

Assay completed in two steps, at the first step the enzyme cocktail for each compound consisted of 50 µl urease enzyme (3 mg/ml in phosphate buffer, pH 7.4), 100 µl of test compound at different concentrations (0–10 mg/ml, in phosphate buffer, pH 7.4) and 850 µl urea solution (30 mM, in phosphate buffer, pH 7.4). This mixture was incubated at 37 °C for 30 min.

For the second step, each 100 µl of the above mixture was added to 500 µl of indicator solutions A (0.5 g phenol and 25 mg sodium nitroprusside in 500 ml distilled water) and 500 µl of B (2.5 g sodium hydroxide and 4.2 ml sodium hypochlorite (5%) in 500 ml distilled water) and further incubated at 37 °C for 30 min. The absorbance of blue-colored indophenol of each cell is related to the percentage of enzyme inhibition using the following equation:

$${\text{I }}\left( \% \right) \, = \, \left[ {{1 } - \, \left( {{\text{T}}/{\text{C}}} \right)} \right] \, \times { 1}00.$$

In this equation, I (%) is assigned to the percent of enzyme inhibition. (T) is assigned to the absorbance of our test compounds and C is assigned to the negative control absorbance which is the absorbance of our cocktail without any inhibitor compound. Thiourea and hydroxyurea were used as the positive controls. The IC_50_ values of test compounds were calculated using GraphPad PRISM 8.0 software (GraphPad, San Diego, CA, USA).

### Antibacterial activity

Two Gram-positive bacteria strains (*Staphylococcus aureus* ATCC 6538 and *Staphylococcus epidermidis* ATCC 12228) and two Gram-negative bacteria strains (*Escherichia coli* ATCC 8739 and *Pseudomonas aeruginosa* ATCC 9027) were used for evaluation of the synthesized *N-*thioacylated ciprofloxacin derivatives **3a**–**n**. All the strains were obtained from Iranian Microbial Collection (Pasteur Institute of Iran, Tehran, Iran). The antibacterial activity of the synthesized compounds was determined according to the agar dilution methods of the National Committee for Clinical Laboratory Standards^43^.

Briefly, a series of twofold dilutions of test compounds **3a–n** and ciprofloxacin, the standard antibiotic, were dissolved in 1 ml of DMSO. Each concentration was added to molten test agars that have been allowed to equilibrate in a water bath to 55 °C, to attain the concentrations ranging from 100 to 0.003 µg/ml. To compare the MIC values; the standard antibiotic, ciprofloxacin was also diluted in the same manner.

To prepare the inoculums suspensions, the bacteria were cultured on Muller-Hinton agar 12–16 h before the test. On the day of the experiment, a single colony of each bacteria was suspended Muller–Hinton broth to reach the turbidity of 0.5 McFarland standard (0.08–0.1 absorbance at 600 nm), which is approximately equivalent to 1.5 $\times$ 10^8^ CFU/ml. The suspensions were then diluted at 1:10 in sterile saline to obtain a concentration of 1.5 $\times$ 10^7^ CFU/ml. The plates were dot inoculated with 2 ul of each bacterial suspension and incubated at 37 °C overnight. The MIC was also defined as the lowest concentration of test compounds that completely prevents the growth of bacteria on agar plate following overnight incubation.

### Anti-ureolytic activity against ureolytic microorganisms

The colorimetric microdilution technique using urea broth media (Merck, supplemented with glucose; pH 6 for *C. neoformans*) was used to determine the ureolytic activity of *C. neoformans* (H99), and clinical isolate of *P. vulgaris* treated with **3n** according to previously reported procedures^18,37^.

### Molecular docking and dynamic simulations

The X-ray crystallographic structure of *JB* urease (www.rcsb.org; PDB ID: 4h9m) and the structure of the compound with the best urease inhibition activity along with the thiourea was used after preparation with the Protein Preparation Wizard and the LigPrep module of Schrödinger platform (Schrödinger, LLC, New York, NY, 2018). Molecular docking evaluations were performed according to previously reported procedures^44^.

The molecular simulation was performed using the Desmond v5.3 (Schrödinger 2018‐4 suite). To build the system for MD simulation, the protein–ligand complexes were solvated with SPC explicit water molecules and placed in the center of an orthorhombic box of appropriate size in the periodic boundary condition. Sufficient counterions and a 0.15 M solution of NaCl were also utilized to neutralize the system and to simulate the real cellular ionic concentrations, respectively. The MD protocol involved minimization, pre-production, and finally production MD simulation steps. In the minimization procedure, the entire system was allowed to relax for 2500 steps by the steepest descent approach. Then the temperature of the system was raised from 0 to 300 K with a small force constant on the enzyme to restrict any drastic changes. MD simulations were performed via NPT (constant number of atoms, constant pressure i.e. 1.01325 bar, and constant temperature i.e. 300 K) ensemble. The Nose–Hoover chain method was used as the default thermostat with 1.0 ps interval and Martyna–Tobias–Klein as the default barostat with 2.0 ps interval by applying isotropic coupling style. Long‐range electrostatic forces were calculated based on the particle‐mesh‐based Ewald approach with the cut‐off radius for Columbia forces set to 9.0 Å. Finally, the system was subjected to produce MD simulations for 30 ns for each protein–ligand complex. During the simulation, every 1000 ps of the actual frame was stored. The dynamic behavior and structural changes of the systems were analyzed by the calculation of the root mean square deviation (RMSD) and RMSF.

### Prime MM-GBSA

The ligand-binding energies (ΔG_Bind_) were calculated using molecular mechanics/generalized born surface area (MM‑GBSA) modules (Schrödinger LLC 2018) based on the following equation:

$$\Delta {\text{G}}_{{{\text{Bind}}}} = {\text{ E}}_{{{\text{Complex}}}} {-} \, \left[ {{\text{E}}_{{{\text{Receptor}}}} + {\text{ E}}_{{{\text{Ligand}}}} } \right],$$

where ΔG_Bind_ is the calculated relative free energy in which it includes both receptor and ligand strain energy. E_Complex_ is defined as the MM-GBSA energy of the minimized complex, and E_Ligand_ is the MM-GBSA energy of the ligand after removing it from the complex and allowing it to relax. E_Receptor_ is the MM-GBSA energy of relaxed protein after separating it from the ligand.

### Prediction of pharmacokinetic properties of synthesis compounds

Prediction of the molecular properties of the synthesized compounds **3a–n** was performed using the online servers as pkCSM (http://biosig.unimelb.edu.au/pkcsm/).

## Supplementary Information

Supplementary Figures.

## Data Availability

The datasets generated and/or analysed during the current study are available in the Worldwide Protein Data Bank (wwPDB) repository (http://www.rcsb.org).
