# Supplementary material for: Design and synthesis of new N-thioacylated ciprofloxacin derivatives as urease inhibitors with potential antibacterial activity
Source: Sci Rep. 2022 Aug 15;12:13827. doi: 10.1038/s41598-022-17993-4 (PMC9378659; doi:10.1038/s41598-022-17993-4)

Fig. S1. 1-cyclopropyl-6-fluoro-4-oxo-7-(4-(phenylcarbonothioyl)piperazin-1-yl)-1,4 dihydroquinoline-3-carboxylic acid ***3a***

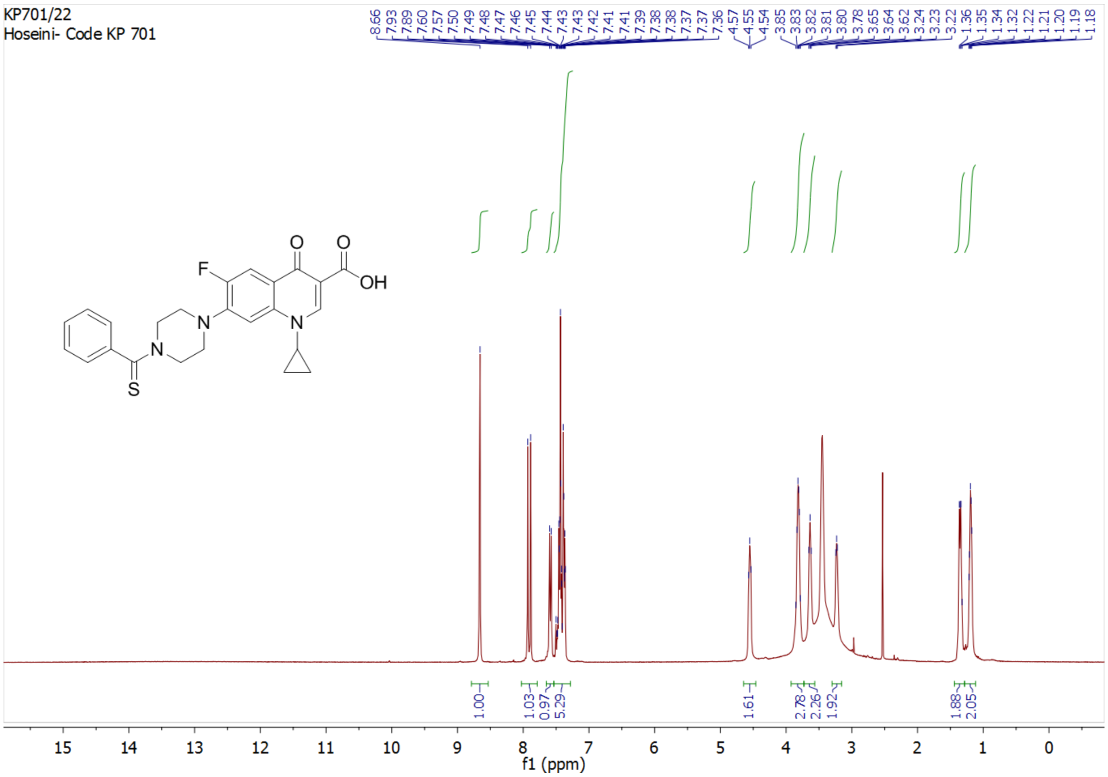

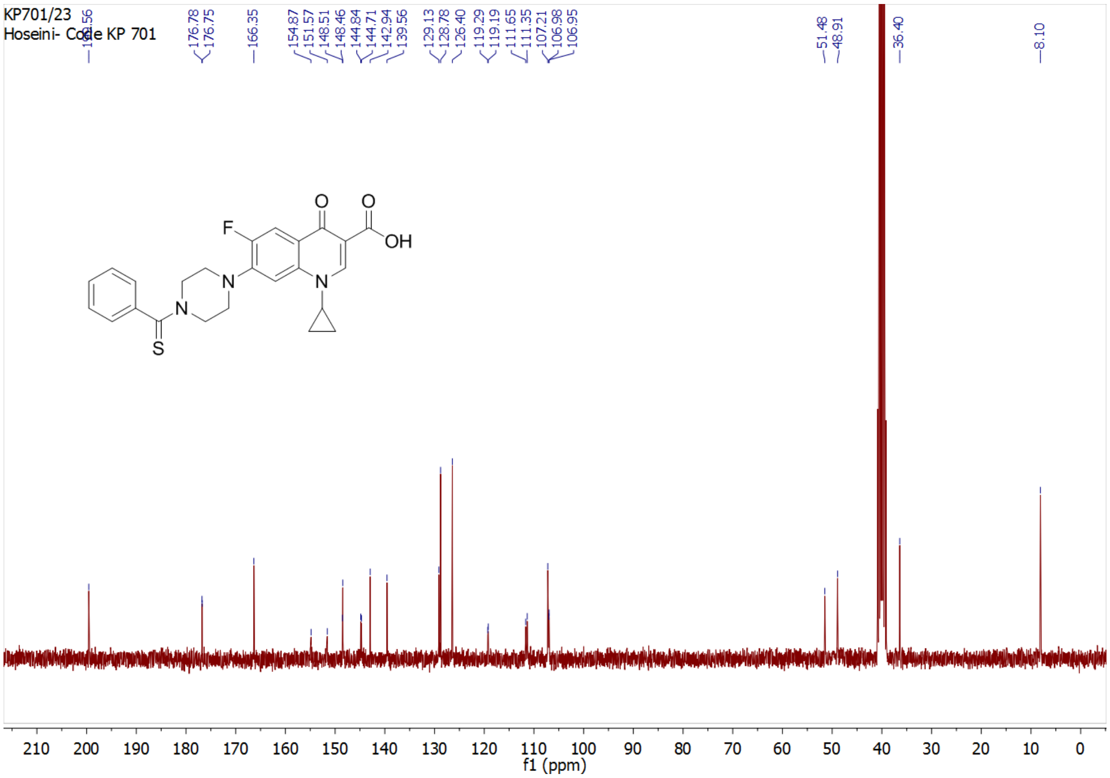

Fig. S2. *1-cyclopropyl-6-fluoro-7-(4-(3-methoxyphenylcarbonothioyl)piperazin-1-yl)-4-oxo-1,4-dihydroquinoline-3-carboxylic acid **3b***

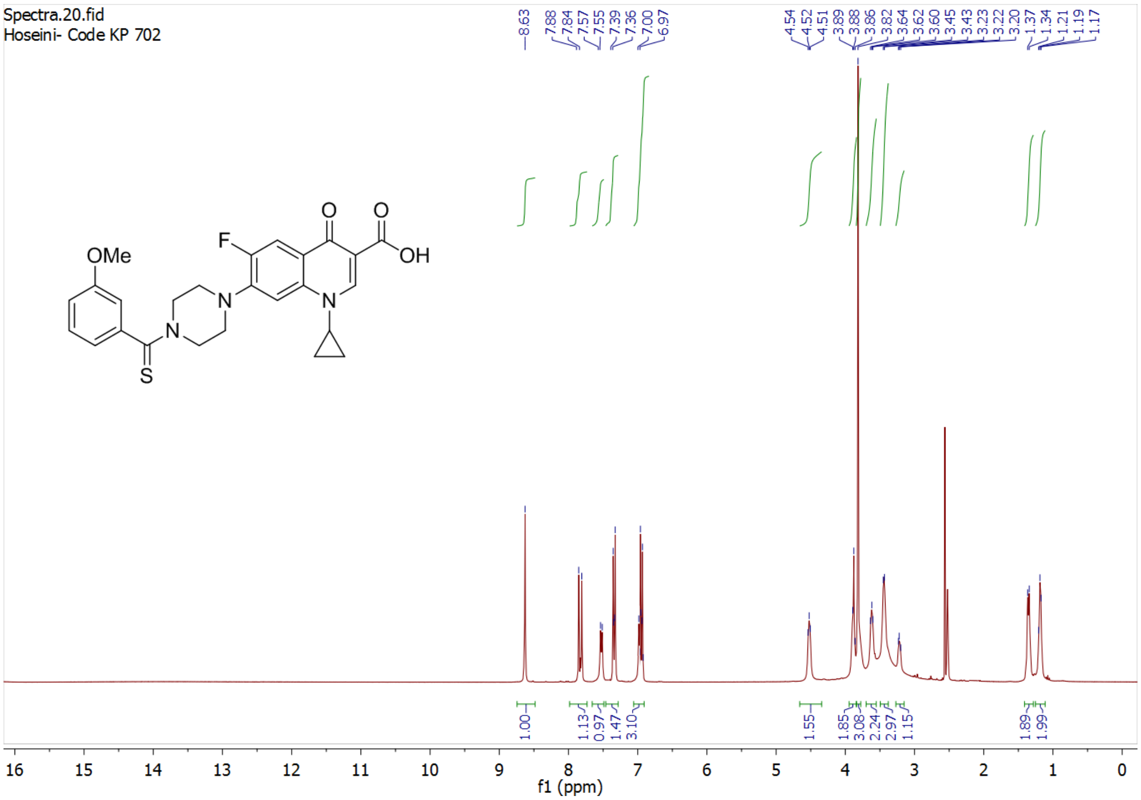

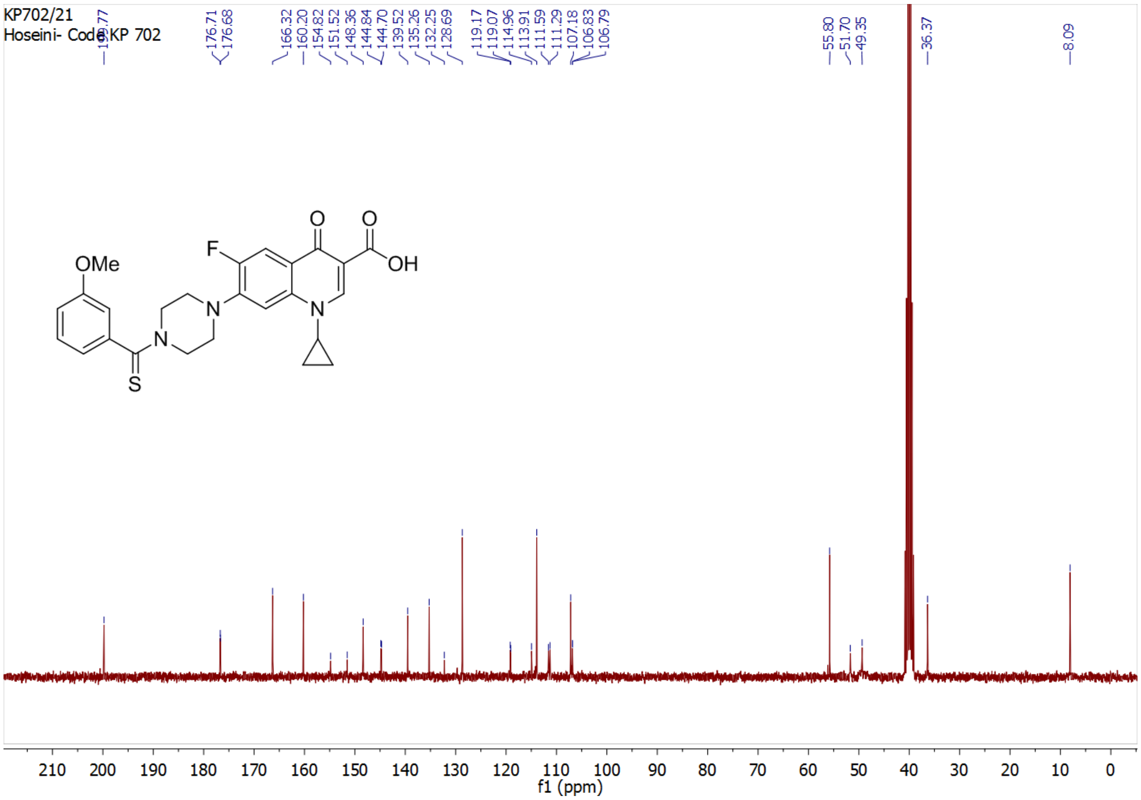

Fig. S3. *1-cyclopropyl-6-fluoro-7-(4-(4-methylphenylcarbonothioyl)piperazin-1-yl)-4-oxo-1,4-dihydroquinoline-3-carboxylic acid* ***3c***

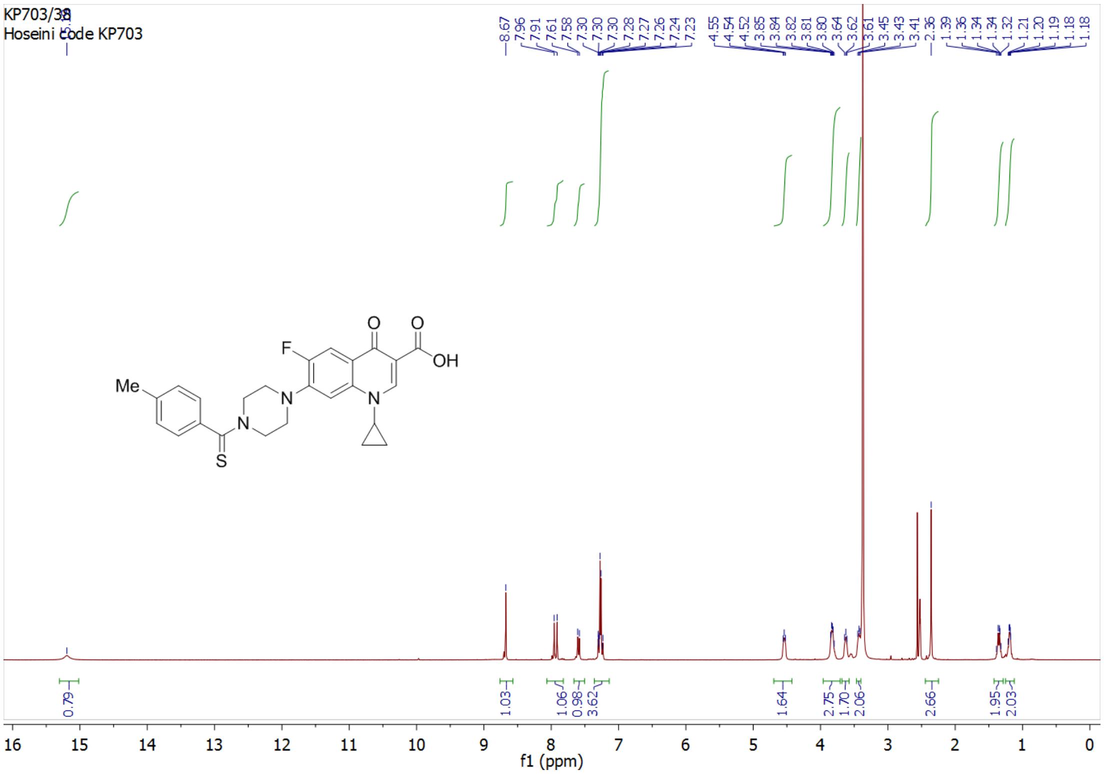

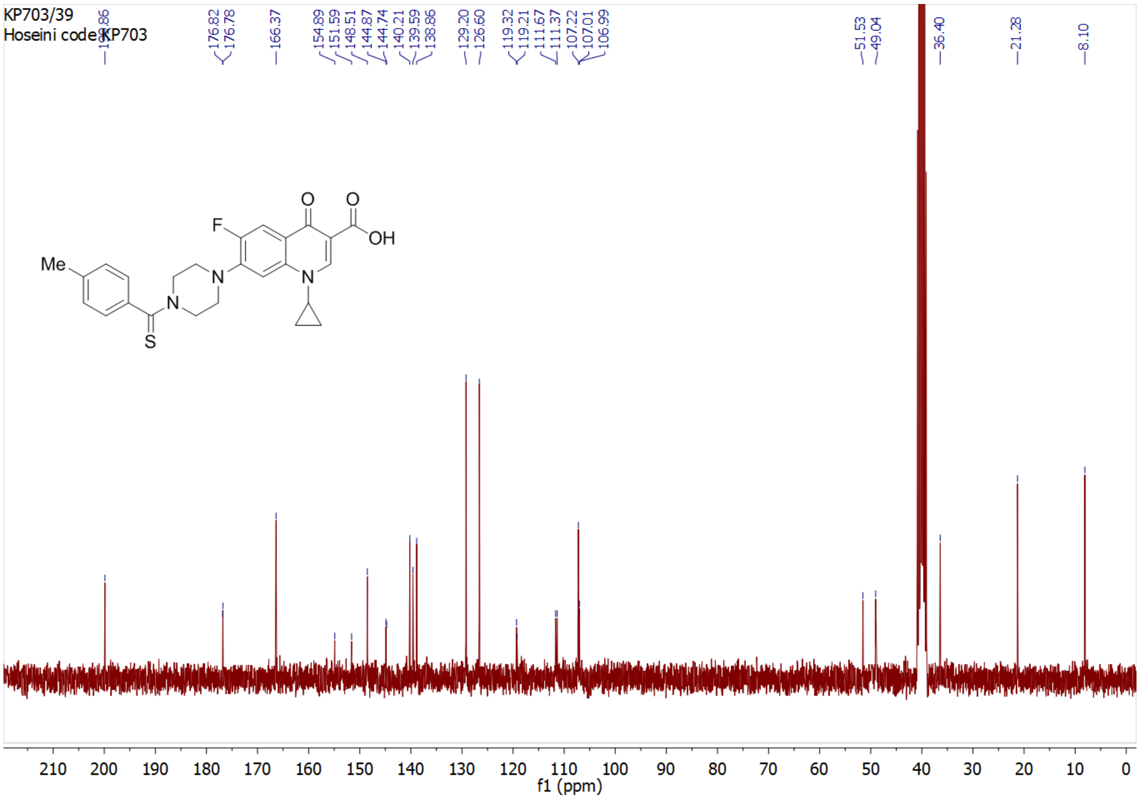

Fig. S4. *1-cyclopropyl-6-fluoro-7-(4-(4-fluorophenylcarbonothioyl)piperazin-1-yl)-4-oxo-1,4-dihydroquinoline-3-carboxylic acid **3d***

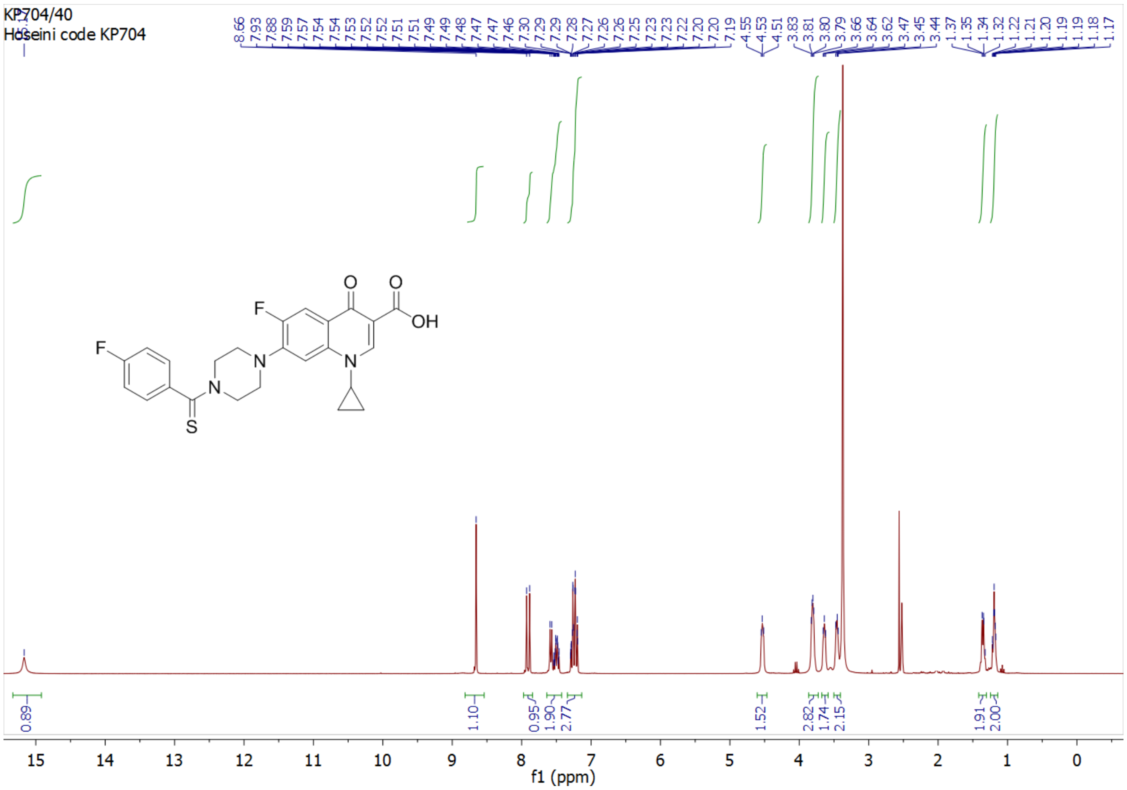

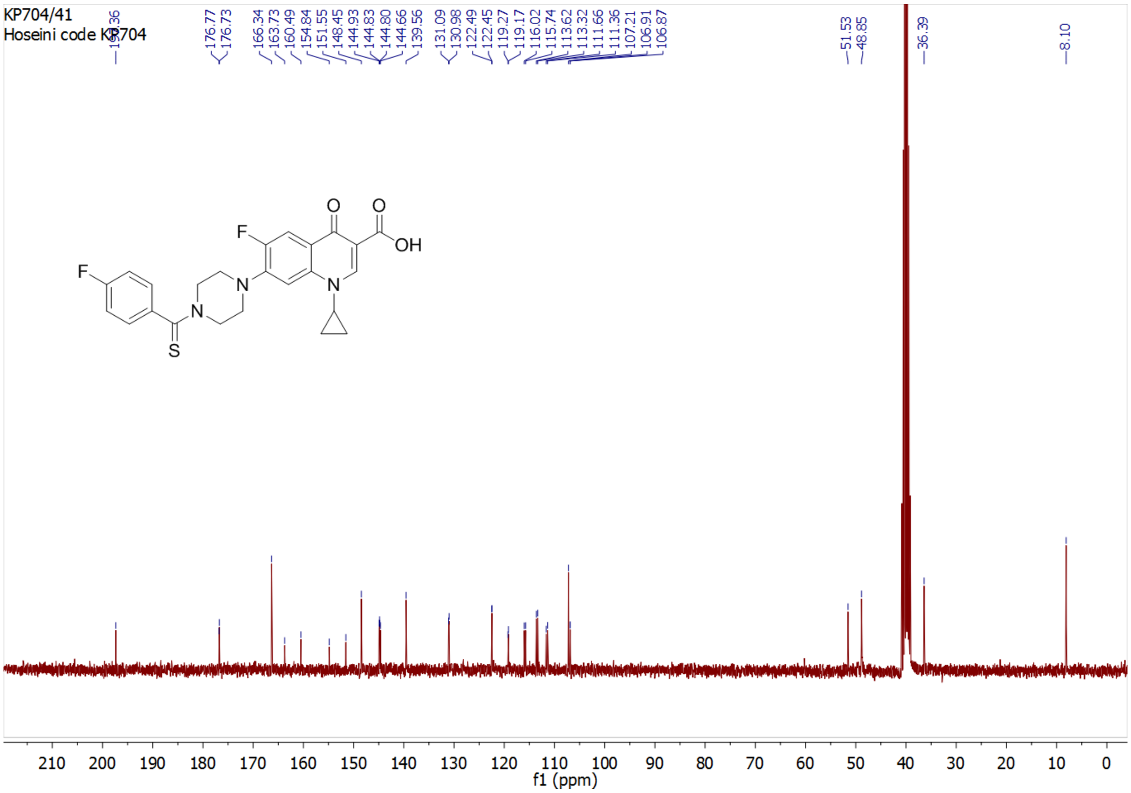

Fig. S5. 7-(4-(4-chlorophenylcarbonothioyl)piperazin-1-yl)-1-cyclopropyl-6-fluoro-4-oxo-1,4-dihydroquinoline-3-carboxylic acid ***3e***

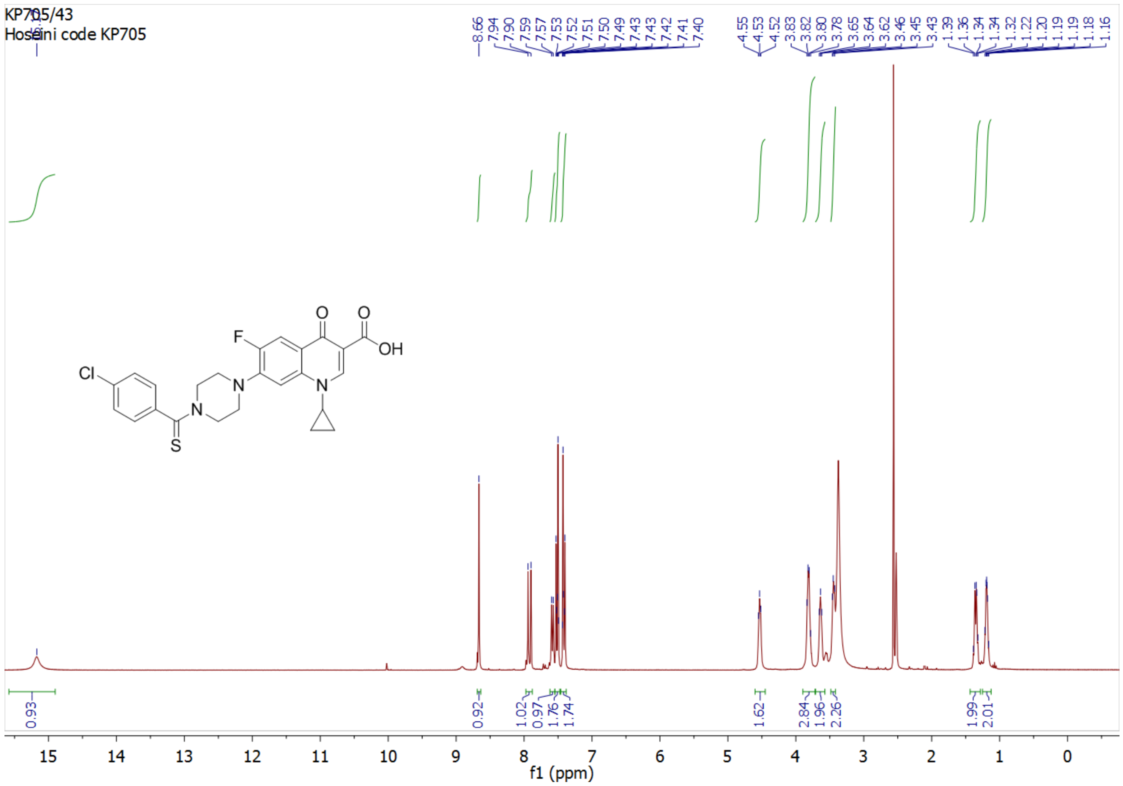

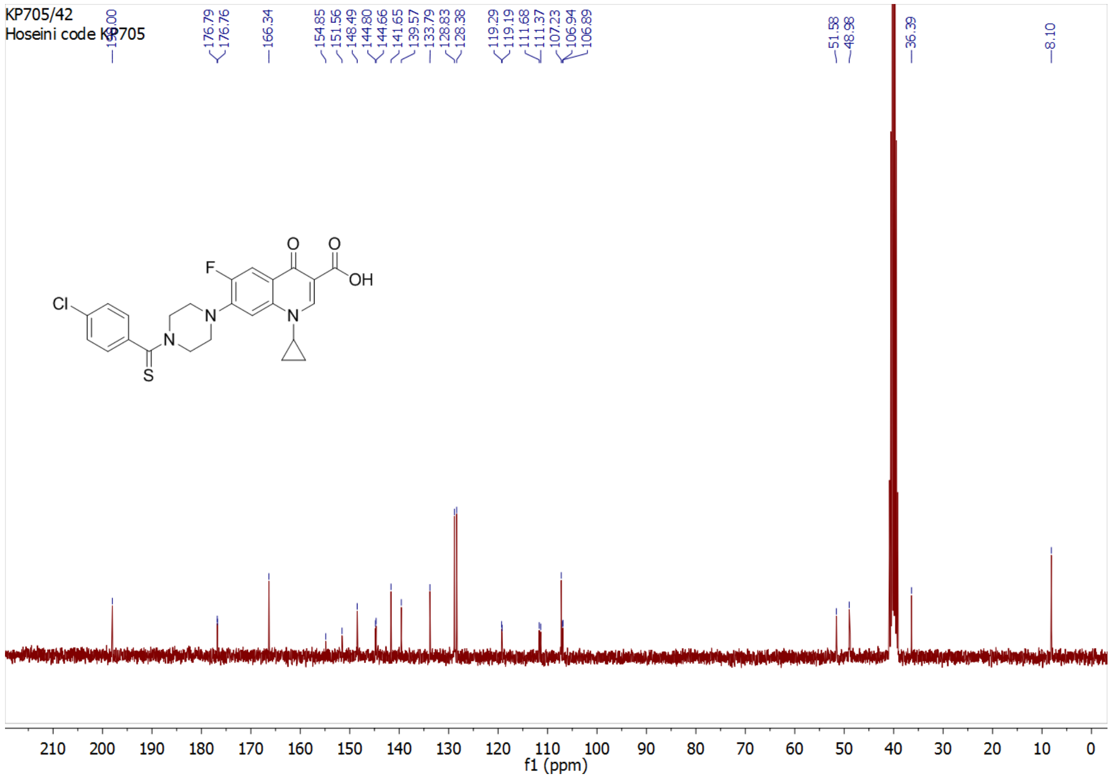

Fig. S6. *1-cyclopropyl-6-fluoro-7-(4-(4-methoxyphenylcarbonothioyl)piperazin-1-yl)-4-oxo-1,4-dihydroquinoline-3-carboxylic acid **3f***

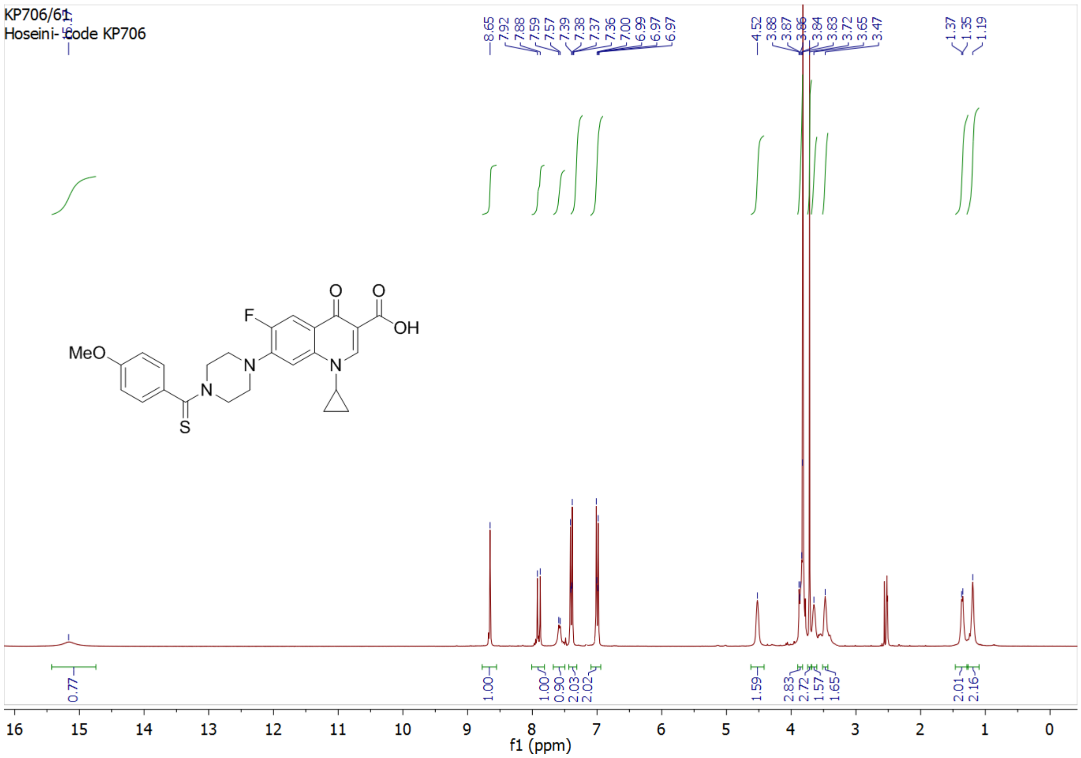

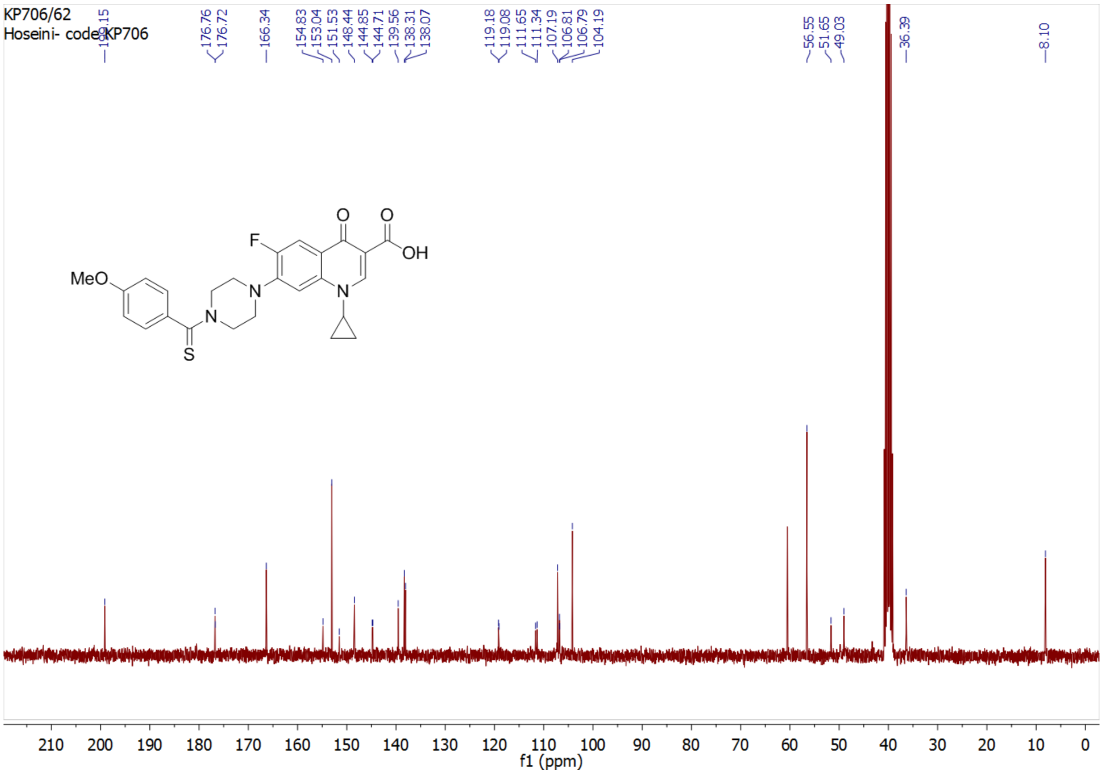

Fig. S7. *1-cyclopropyl-6-fluoro-7-(4-(3-nitrophenylcarbonothioyl)piperazin-1-yl)-4-oxo-1,4-dihydroquinoline-3-carboxylic acid* ***3g***

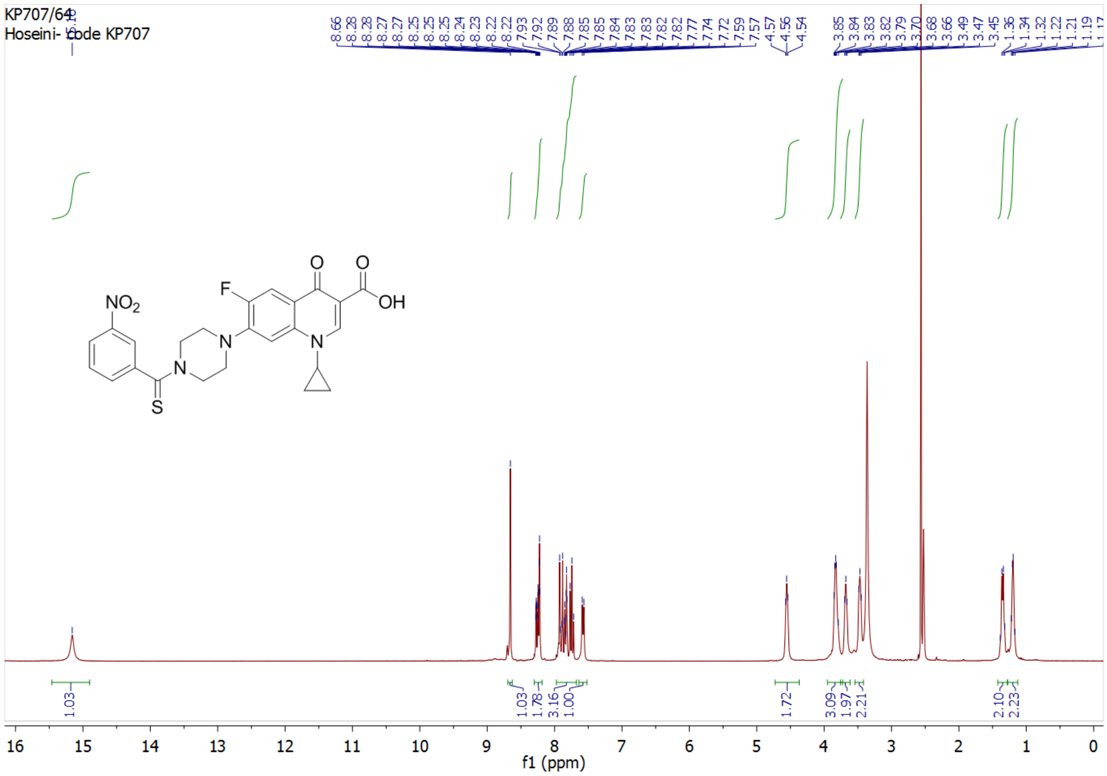

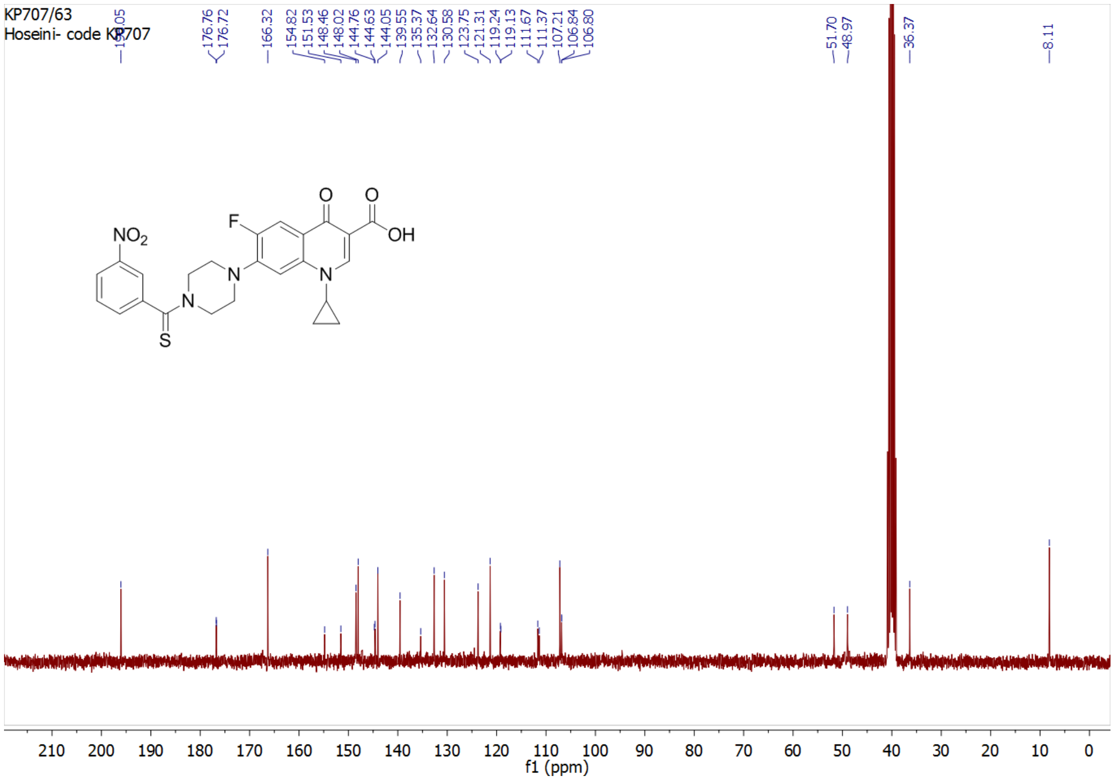

Fig. S8. *1-cyclopropyl-6-fluoro-7-(4-(3-fluorophenylcarbonothioyl)piperazin-1-yl)-4-oxo-1,4-dihydroquinoline-3-carboxylic acid* ***3h***

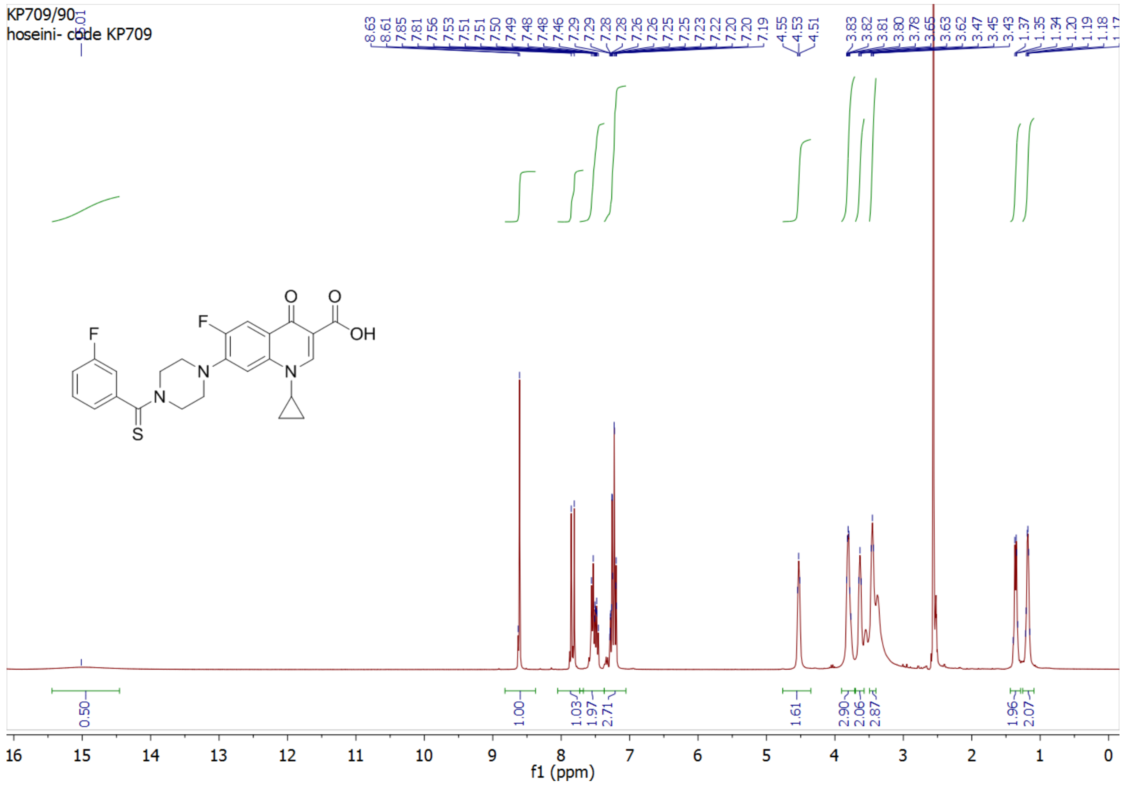

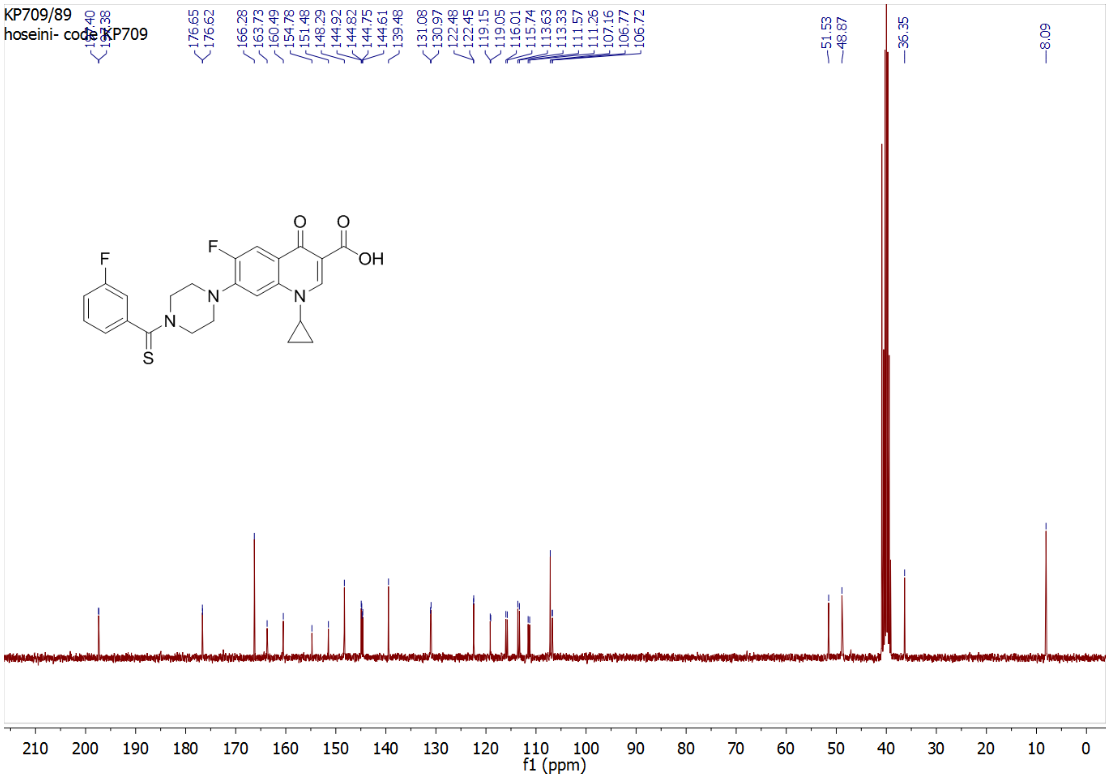

Fig. S9. *1-cyclopropyl-6-fluoro-7-(4-(2-fluorophenylcarbonothioyl)piperazin-1-yl)-4-oxo-1,4-dihydroquinoline-3-carboxylic acid **3i***

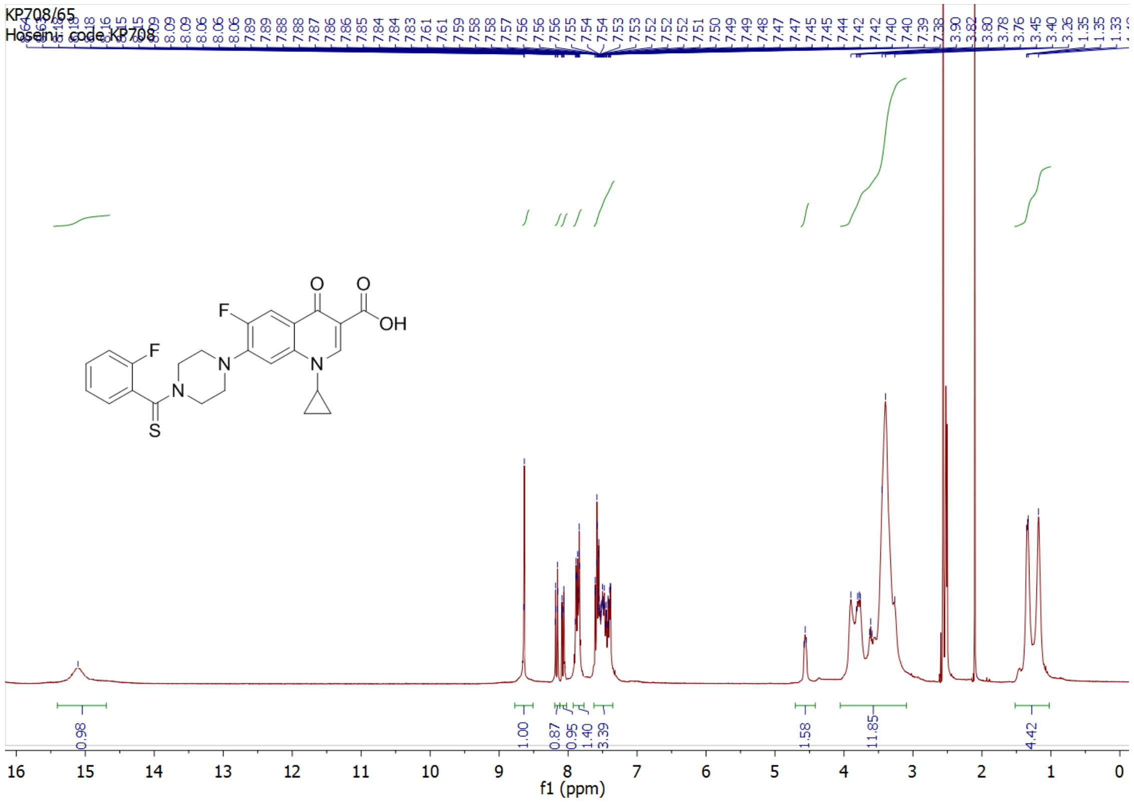

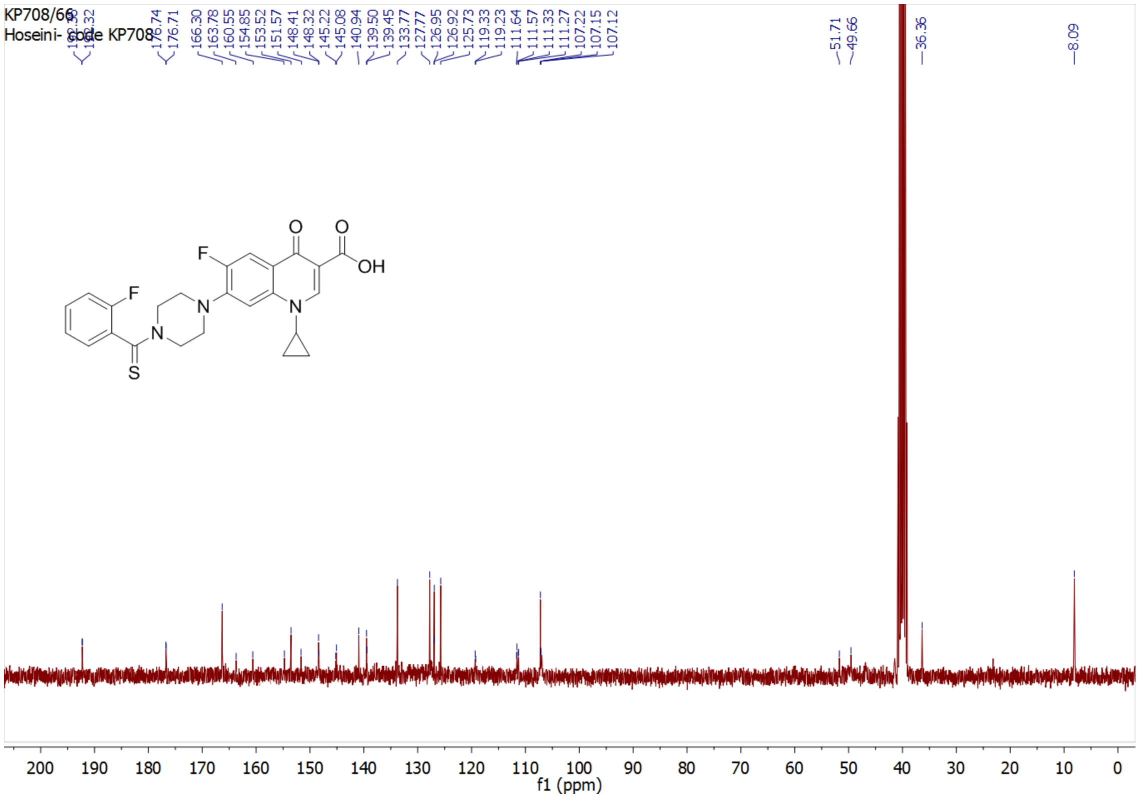

Fig. S10. *1-cyclopropyl-6-fluoro-7-(4-(3-hydroxyphenylcarbonothioyl)piperazin-1-yl)-4-oxo-1,4-dihydroquinoline-3-carboxylic acid* ***3j***

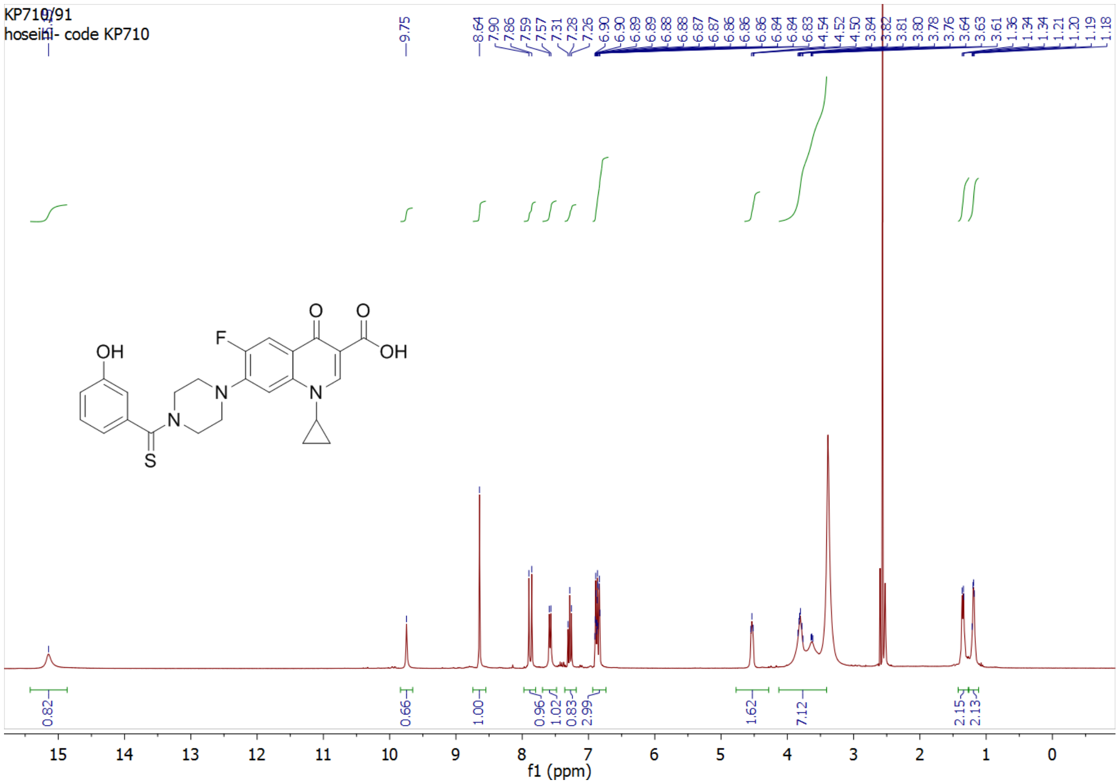

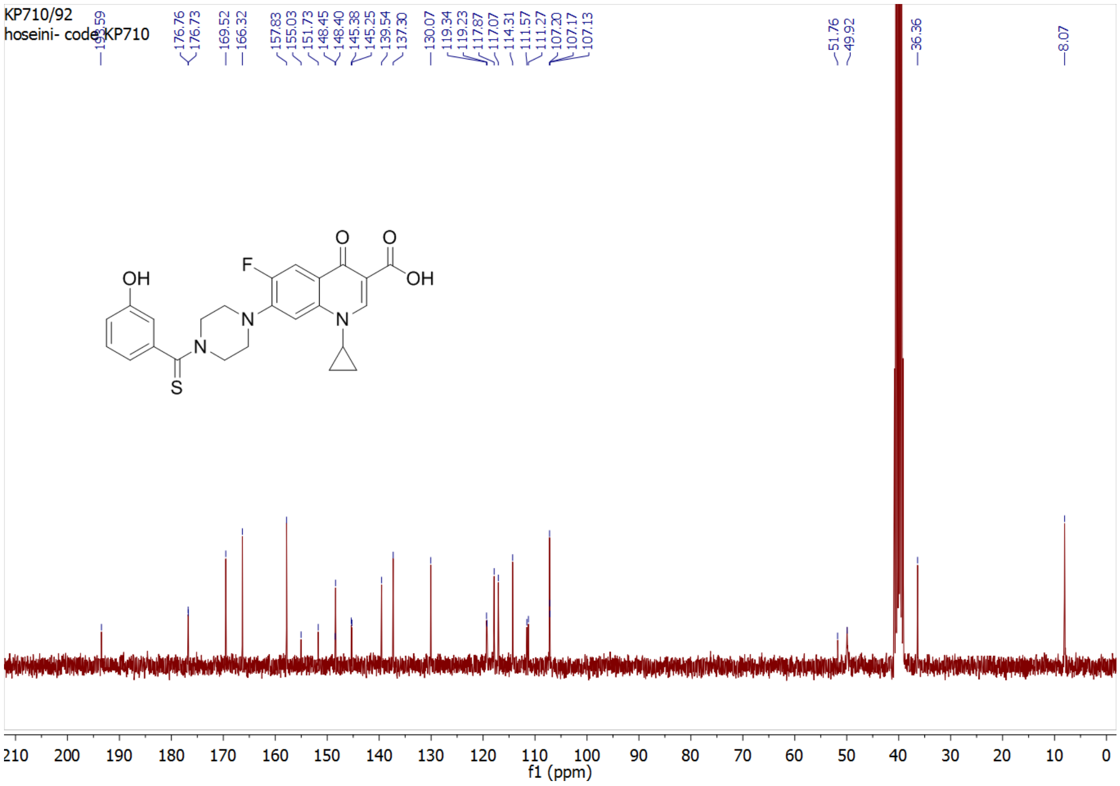

Fig. S11. *1-cyclopropyl-6-fluoro-4-oxo-7-(4-(3-phenoxyphenylcarbonothioyl)piperazin-1-yl)-1,4-dihydroquinoline-3-carboxylic acid* ***3k***

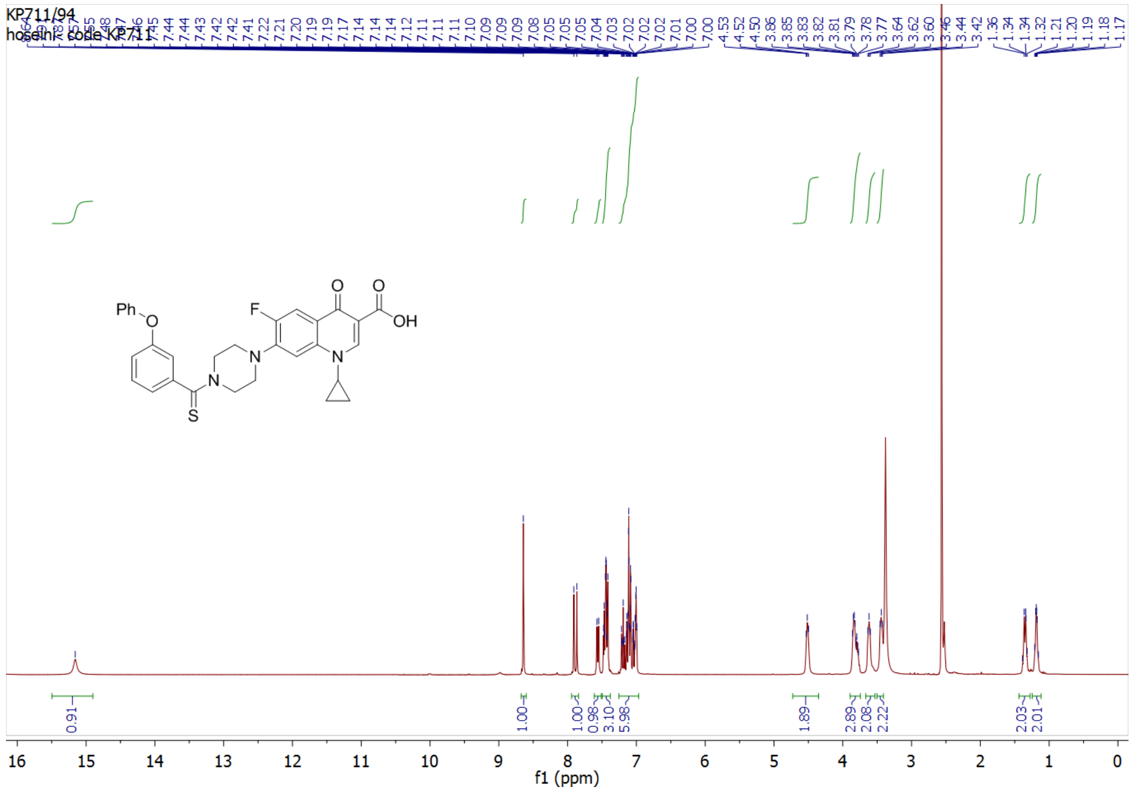

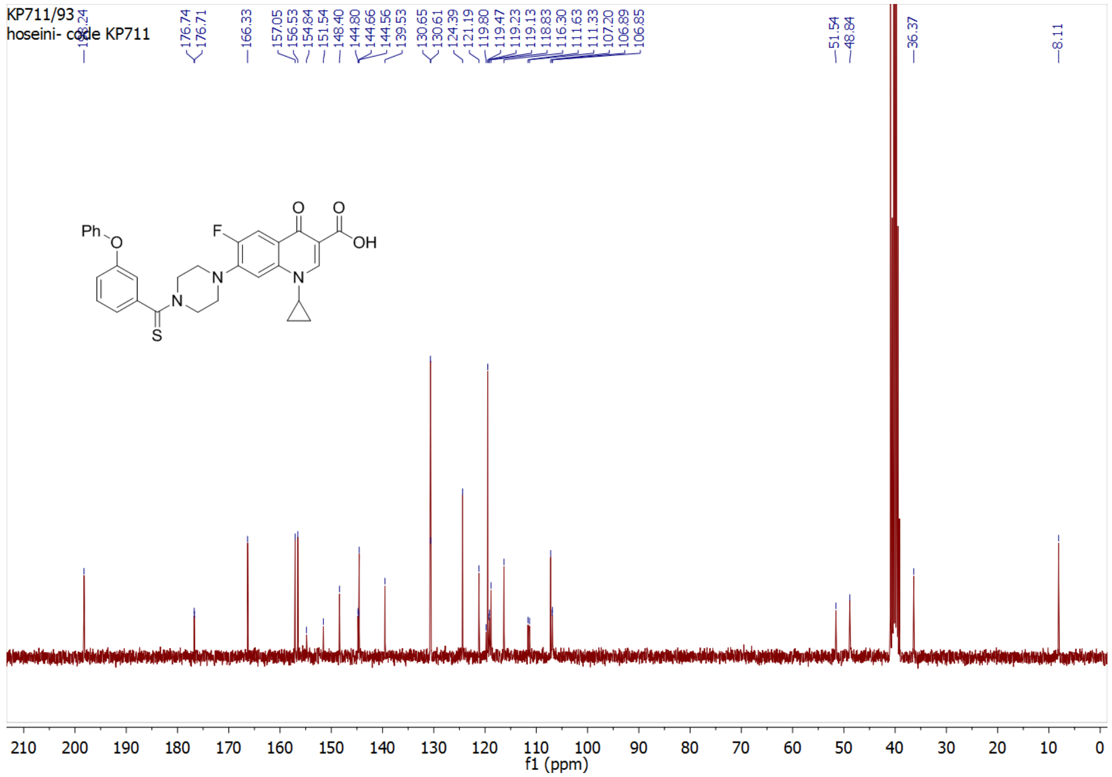

Fig. S12. *7-(4-(4-bromophenylcarbonothioyl)piperazin-1-yl)-1-cyclopropyl-6-fluoro-4-oxo-1,4-dihydroquinoline-3-carboxylic acid* ***3l***

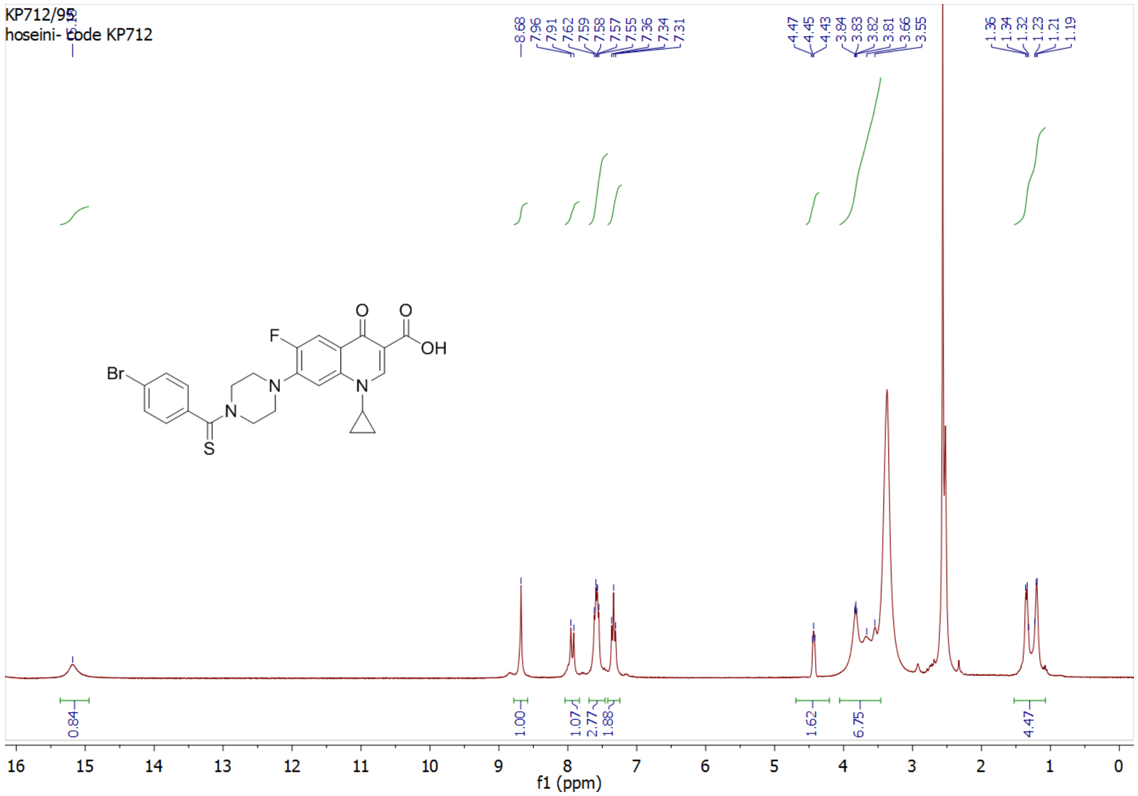

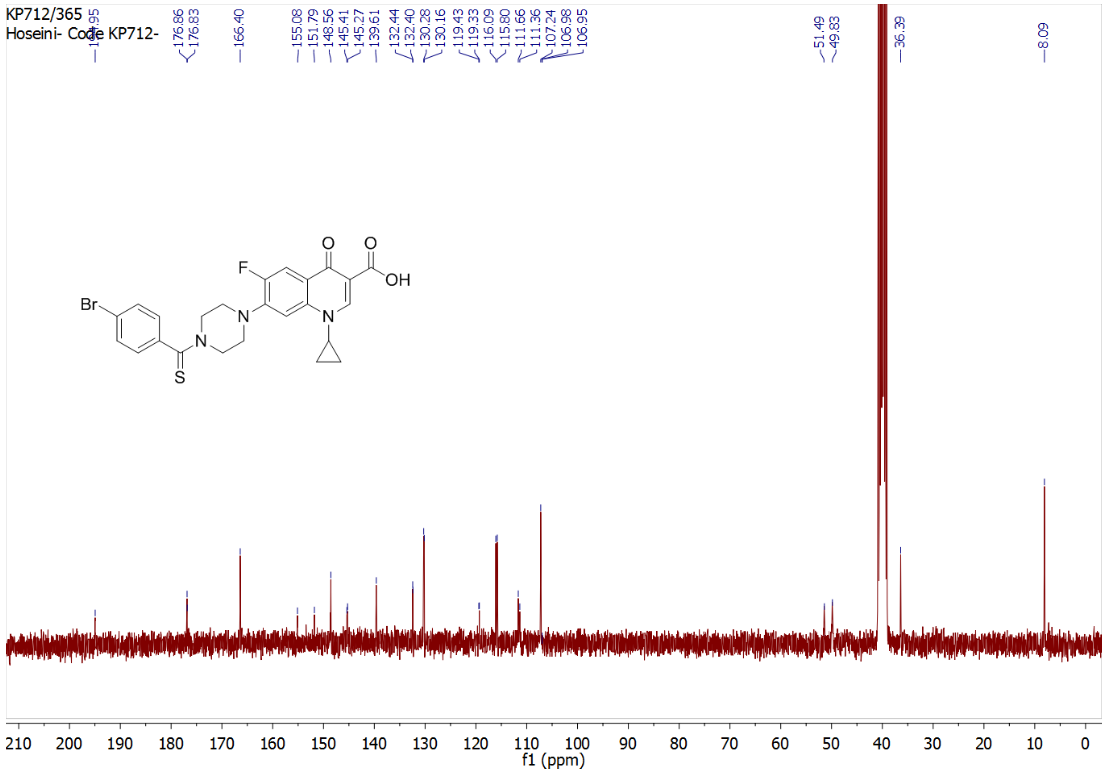

Fig. S13. *7-(4-(3-chlorophenylcarbonothioyl)piperazin-1-yl)-1-cyclopropyl-6-fluoro-4-oxo-1,4-dihydroquinoline-3-carboxylic acid* ***3m***

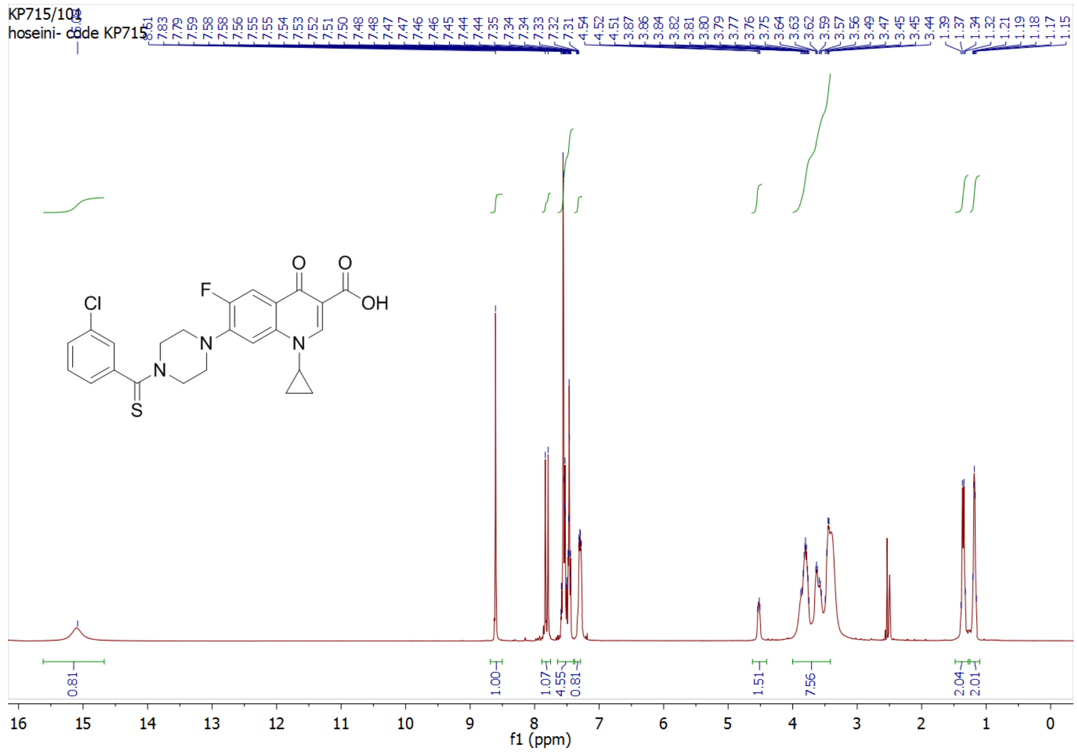

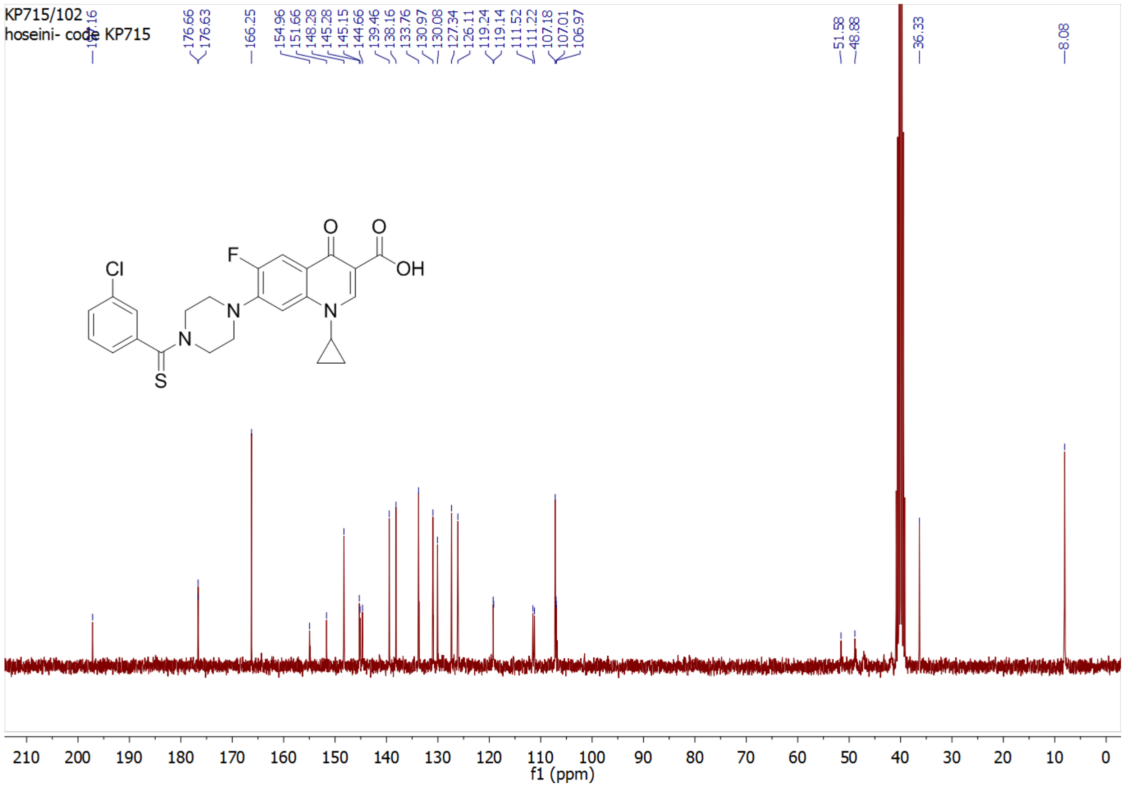

Fig. S14. *1-cyclopropyl-6-fluoro-7-(4-(4-nitrophenylcarbonothioyl)piperazin-1-yl)-4-oxo-1,4-dihydroquinoline-3-carboxylic acid* ***3n***

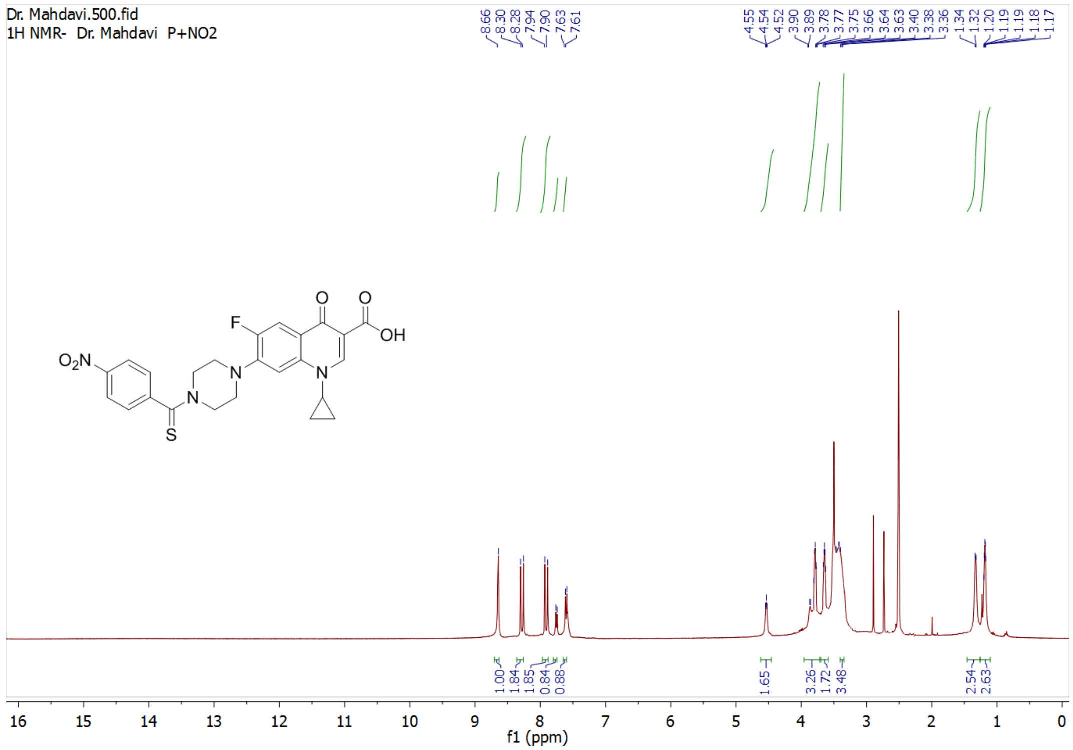

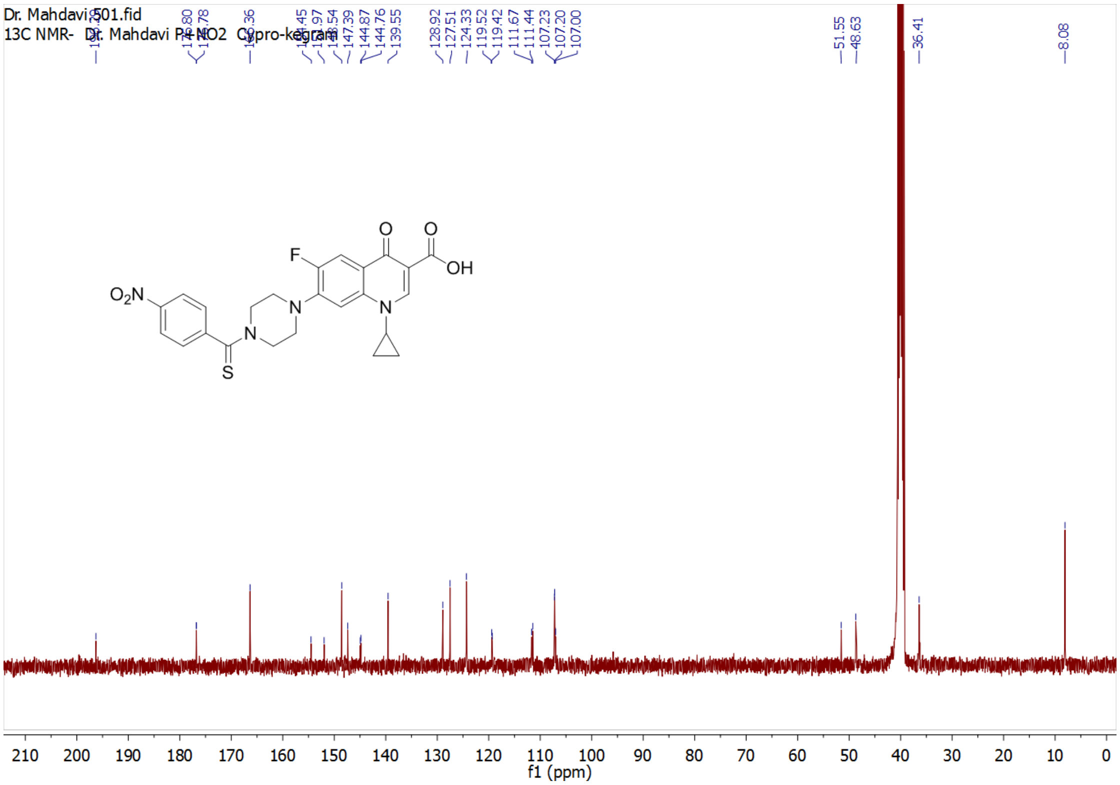

Supplement: Supplementary file 1 — Supplementary Figures. [file 41598_2022_17993_MOESM1_ESM.pdf]
